# Depth-enhanced molecular imaging with two-photon oblique plane microscopy

**Published:** 2025-11-12

**Authors:** Kevin Keomanee-Dizon, Yaakov Clenman, Alejandra Duran, Sergey Ryabichko, Pauline Hansen, Tohn Borjigin, Richard Thornton, Jared E. Toettcher, Harold M. McNamara

**Affiliations:** 1 Joseph Henry Laboratories of Physics, Princeton University, Princeton, NJ 08544, USA; 2 Lewis-Sigler Institute for Integrative Genomics, Princeton University, Princeton, NJ 08544, USA; 3 Department of Molecular Biology, Princeton University, Princeton, NJ 08544, USA; 4 Omenn-Darling Bioengineering Institute, Princeton University, Princeton, NJ 08544, USA

## Abstract

High-numerical-aperture (NA) oblique plane microscopy enables noninvasive fluorescence imaging of subcellular dynamics without requiring radical sample modification. However, performance degrades at depth in multicellular specimens as scattering and refractive-index heterogeneity raise out-of-focus background. We report a two-photon oblique plane microscope that improves resolution at depth by combining high-NA single-objective detection with multiphoton plane illumination. The microscope achieves ~300 nm lateral and ~650 nm axial resolution, with single-molecule sensitivity *in vivo*. Compared with two-photon point scanning, the lower illumination NA delivers an order of magnitude lower peak intensity, enabling >5× faster volumetric acquisition (up to 3.25 × 10^6^ voxels s^−1^) with reduced photodamage. In multicellular contexts, near-infrared nonlinear excitation enhances contrast throughout the illumination depth by ~2× and restores volumetric resolving power by > 2× relative to linear excitation. We demonstrate these capabilities through molecular imaging of epithelial tissue, stem-cell-derived gastruloids, and living fruit fly embryos, including multicolor transcription-factor dynamics, optogenetic subcellular control, and single-mRNA tracking, all using standard glass-based mounting.

Light-sheet microscopy offers a crucial window into the intrinsic complexity of the living world, from single-molecule dynamics in cells [1] to whole-brain neural activity in behaving animals [2, 3]. By restricting illumination to the vicinity of the detection depth of field, it permits fast, plane-wise acquisition with high contrast, inherent optical sectioning, and low photodamage. However, most implementations rely on multi-objective geometries and custom sample holders that are incompatible with standard glass-based mounting protocols [4]. Single-objective implementations, such as oblique plane microscopy (OPM) [5, 6], preserve standard sample mounting while enabling high-NA (>1) detection via refractive-index-mismatched remote focusing [7–9]. In multicellular specimens, scattering and refractive-index heterogeneity deform and thicken one-photon (1P) light sheets. Wide-field detection then integrates out-of-plane fluorescence, degrading contrast, spatial resolution, and the fidelity of weak molecular signals.

Two-photon (2P) excitation helps bypass these limits: longer wavelengths scatter less, and the quadratic nonlinearity spatially concentrates fluorescence to the focal volume, suppressing background and improving sectioning at depth [10–12]. To date, most multiphoton imaging remains scanning-based, collecting signal from a point [13], a line [14], or multiple simultaneous foci [15], which limits speed. For fast point or line scanning, increasing the voxel rate by N shortens the dwell to $\tau^{\prime }=\tau /N$, reducing photons per voxel at fixed average power at the specimen $P_{\text{s}}$. Since 2P signal $S\propto P_{\text{s}}^{2}\tau$, maintaining the signal-to-noise ratio (SNR) requires $P_{\text{s}}^{\prime }\approx \sqrt{N}P_{\text{s}}$. In multifocal 2P, an N-fold speedup with N simultaneous foci requires $P_{\text{s}}^{\prime }\approx NP_{\text{s}}$ to maintain per-voxel SNR; otherwise it falls as $\sim 1/N^{2}$ [16]. While SNR is fundamentally constrained by fluorophore photophysics and shot noise, in practice linear heating (∝ average power) and higher-order nonlinear photodamage (∝ peak intensity) make fast, gentle volumetric imaging at subcellular resolution challenging for common 2P methods.

Multiphoton plane illumination offers surprising advantages in both speed and noninvasiveness [16, 17]. First, orthogonal illumination parallelizes excitation along the propagation direction: N voxels in the plane are excited and read out simultaneously on the camera; at fixed voxel rate the per-voxel dwell equals the exposure time (not τ/N as in fast scanning with a point detector) [16]. This exposure can be orders of magnitude longer than the equivalent point-scanning dwell, proportionally increasing photon accumulation at the same average power. Second, because axial resolution in light-sheet imaging is set by the sheet thickness convolved with the detection depth of field (not a tight 3D focus), the illumination NA can be reduced by α≈5-10 while preserving the point-scan axial point-spread function (PSF), which lowers the peak (instantaneous) intensity by $\alpha^{2}$ [17]. Since 2P signal scales as $S\propto I_{\text{peak}}^{2}\tau$, with $I_{\text{peak}}\propto {\text{NA}}^{2}$, this NA reduction depresses signal by $\alpha^{4}$. Compensating at constant SNR, the per-voxel dwell can increase by $\alpha^{4}$ (~6 × 10^2^–10^4^ longer) while the required peak intensity drops by $\alpha^{2}$ (~25–100× lower) [16, 17]. Lower peak intensity directly suppresses higher-order (>2P) nonlinear processes that dominate phototoxicity *in vivo* [18], allowing higher tolerable average powers and thus improved SNR and volumetric imaging speed without crossing photodamage thresholds [17, 18].

High-resolution 2P light-sheet systems have been demonstrated [2, 17, 19, 20], but use multi-objective geometries that complicate sample mounting and restrict sample size. Single-objective 2P light-sheet variants support conventional mounting but operate at low detection NA (~0.34) [21], preventing sub-μm resolution and sufficient photon collection for single-molecule sensitivity.

To overcome these inherent optical and sample-handling tradeoffs, we developed 2P oblique plane microscopy (2P-OPM), combining high-NA single-objective detection with multiphoton plane illumination. Refractive-index mismatch in the remote-focus space provides high-NA detection and a conventional objective-sample interface [7–9, 22], permitting high-resolution imaging through common glass substrates. By delivering a near-infrared (NIR) 2P light sheet through the same objective used for collection, 2P-OPM better confines excitation to the detection depth of field and retains robust volumetric resolution at depth compared to 1P. Our system achieves raw resolutions of ~300 nm laterally and ~650 nm axially, the highest for multiphoton OPM to date. The resulting gains in resolution, contrast, and sectioning improve low-SNR performance and enable single-molecule sensitivity *in vivo*. We show simultaneous multicolor imaging, optogenetic control and measurement of subcellular organization, and volumetric acquisition more than fivefold faster than traditional 2P point scanning. These capabilities are demonstrated by molecular imaging across a broad range of multicellular contexts, from gastruloids to transcriptional dynamics in living fly embryos, all prepared with standard mounting.

## RESULTS

### High-NA OPM with 2P excitation

**FIG. 1.**
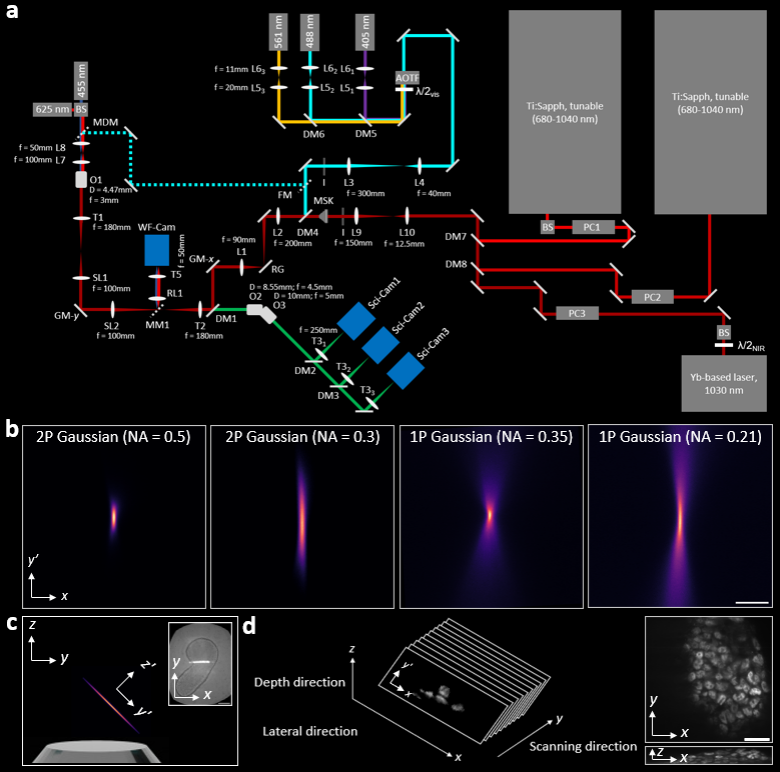
Two-photon oblique plane microscopy (2P-OPM). **(a)** Optical microscope schematic. Two Ti:sapphire and one ytterbium-based laser provide 2P excitation (red path), modulated by Pockels cells (PC1-PC3). NIR beams are combined via dichroic mirrors (DM7-DM8), expanded to 12 mm, and passed through an iris (I) to set illumination NA. A layer-cake phase mask (MSK) can optionally generate a Bessel-like focus. CW lasers at 405 nm (purple), 488 nm (blue), and 561 nm (yellow) provide 1P excitation. These beams are collimated and expanded (L5_1–3_ and L6_1–3_) to 1.3 mm, combined via DM5 and DM6, modulated with a half-wave plate ($\lambda /2_{\text{VIS}}$) and acousto-optic tunable filter (AOTF), and expanded 7.5× (L3-L4). I sets the illumination NA before merging with the NIR path at DM4 (blue path). The combined beam is focused by L2 onto a resonant galvo (RG), relayed to scanning galvos GM-x and GM-y, and then through L1, scan lenses SL2 and SL1, and tube lenses T2 and T1 to the rear focal plane of the primary objective O1. DM1 directs light obliquely into the specimen. Fluorescence is collected by O1 and relayed by remote objectives O2 and O3 to form an image. DM2, DM3, and a broadband mirror split fluorescence emission into three channels, focused onto Sci-Cam1–3 by T3_1–3_. Flip mirror (FM) directs excitation to a transmitted illumination mode with a movable dichroic mirror (MDM) and lenses L7–L8. Removing MDM permits a beam splitter (BS) to inject 455 nm and/or 625 nm LED light for bright-field illumination. Movable mirror MM1 diverts detected light to a wide-field inspection camera (WF-Cam) via relay lens R1 and T5. Complete details on the optical path are given in Methods; key parts are given in Table I; see also Fig. S1. **(b)** Experimental images of fluorescence excited by 2P Gaussian beams at NA 0.5 (column 1) and NA 0.3 (column 2), and 1P Gaussian beams at NA 0.35 (column 3) and NA 0.21 (column 4), which are rapidly swept in the x direction to create light sheets. See Fig. S7 for intensity profiles. Scale bar, 2μm. **(c)** A 2P light sheet is launched obliquely at 45° from O1. The $y,z,y^{\prime }$ and $z^{\prime }$ directions are indicated. 2D data acquired by collecting fluorescence (light cones not shown) with the same objective. Inset: transmitted light image of a 120-hr gastroloid with the light sheet overlaid. Scale bar, 100μm **(d)** Scanning GM-y in y sequentially acquires oblique 2D light-sheet images, with width x, length $y^{\prime }$, and depth z to form a 3D volume. Inset: xy (top) and xz (bottom) orthoslices of the 3D reconstructed gastruloid from the inset in (c). See also Methods and Fig. S14. Scale bar, 20μm.

Our design starts with high-NA OPM to enable high-resolution imaging through standard glass substrates [7–9, 22]. Three imaging subsystems are arranged in series so that the specimen is selectively illuminated with an oblique sheet through the same objective (O1) used for collection (Fig. 1a,c). Fluorescence is relayed by secondary (O2) and tertiary (O3) objectives and focused onto a camera (Fig. 1a and S1). This single-objective geometry provides flexibility in sample mounting similar to traditional microscopy, accommodating large samples as well as standard glass slides and plates. High-NA OPM harnesses a deliberate refractive-index mismatch between O2 and O3 that compresses the light cone, preserving the large effective detection NA of O1 and yielding higher spatial resolution and photon-collection efficiency than dual-objective configurations [7–9]. In practice, light throughput and sensitivity is limited by losses in the relay and remote-focus optics.

To mitigate these losses and broaden wavelength bandwidth (for both 1P and 2P operation), we use Plössl lenses instead of thick scan lenses [23], both upstream and downstream of our y-scanning galvo mirror (GM-y; Fig. 1a), used to sequentially acquire 2D images and assemble 3D volumes (Fig. 1d) [7, 21]. Each Plössl lens is composed of a pair of broadband double achromats opposing each other with a small (~2 mm) air gap. We also use double achromats as tube lenses T3_1–3_ with a focal length f=250mm, yielding an overall system magnification of 75×. This properly samples the diffraction-limited resolution according to the Nyquist criterion. Three sC-MOS cameras (Sci-Cam1–3) receive spectrally separated emission by a dichroic-mirror tree (Fig. 1a), enabling simultaneous multicolor imaging: Sci-Cam1 collects 420–474 nm; Sci-Cam2 collects 502–548 nm; Sci-Cam3 collects 579–620 nm and 662–734 nm (Fig. S4). With a 45° tilt relative to O1, the effective theoretical detection NA is 1.44 along the x direction and 1.29 along the tiltlimited y direction (Fig. S2). Transmission through our detection path (power at T3 relative to T1, excluding the dichroic-mirror tree) is ~60% at 488 nm, an improvement of ~20 percentage points over the prior laser-scanning high-NA OPM design [9].

To extend the imaging depth of high-NA OPM, we employ 2P light-sheet excitation with ultrafast NIR laser pulses (Methods and Table I). Compared to the visible light used in 1P, the longer NIR wavelengths scatter less, and the quadratic dependence of 2P-excited fluorescence signal on the excitation intensity confines fluorescence near the focus, maintaining optical sectioning even when the sheet broadens by scattering [2]. We generate 2P light sheets by sweeping a beam focus with a pupil-conjugate x-galvo (GM-x) across the detection focal plane to form an oblique $xy^{\prime }$ plane (Fig. 1a–d); a sample-conjugate resonant galvo (RG) “wobbles” the sheet to suppress shadowing artifacts from absorption or scattering (Fig. S1) [24]. The focus is swept along the in-plane axis x over 100-200μm (chosen per sample to avoid edge-scanning artifacts). Sweeping the beam in time keeps the instantaneous intensity at the focus high at each position, while redistributing it along x over the exposure; the camera integrates these passes into an effectively uniform sheet. By contrast, a static sheet of length x distributes power over all x simultaneously, imposing a $1/x^{2}$ per-voxel penalty and a 1/x plane-integrated loss (at fixed average power), whereas a swept focus avoids the $1/x^{2}$ per-voxel penalty and keeps the plane-integrated 2P signal ~constant with x [17].

Our instrument operates in both 2P and 1P modes (Fig. 1a and S1), enabling direct quantitative comparison on the same biological sample. In either mode, a pupil-conjugate adjustable iris (I) sets the illumination NA and thus controls the light-sheet thickness and propagation length. For 2P, we typically use NAs of 0.3–0.5 to match the axial resolution of conventional 2P point scanning at NA ≈ 1.29 (Fig. 1b and S7). At matched voxel rate and axial resolution, this NA reduction permits the per-voxel dwell to increase by $\alpha^{4}$, corresponding to ~45–340× longer dwell for our NA range at fixed per-voxel SNR. Because the peak intensity scales as $I_{\text{peak}}\propto {\text{NA}}^{2}$, a 2.6–4.3× reduction in illumination NA lowers peak intensity by 7–19× at equal average power. We evaluated propagation-invariant, Bessel-like beams [25, 26] and found a ~3× longer usable sheet while maintaining a thin ~0.5μm waist (Methods; Figs. S5–S7, S11). Despite the extended field, we favored Gaussian sheets for bioimaging because concentrating power in the main lobe (no side lobes) produces a higher signal rate at a given average power.

A transmitted wide-field mode (WF-Cam) provides low-magnification view-finding and macroscopic global context, complementing high-resolution fluorescence imaging (Fig. 1c,d and S8). It also serves as a label-free photoperturbation indicator, with bright-field imaging offering a simple readout of specimen health (Video 1).

### Point spread function and resolution benchmarking

**FIG. 2.**
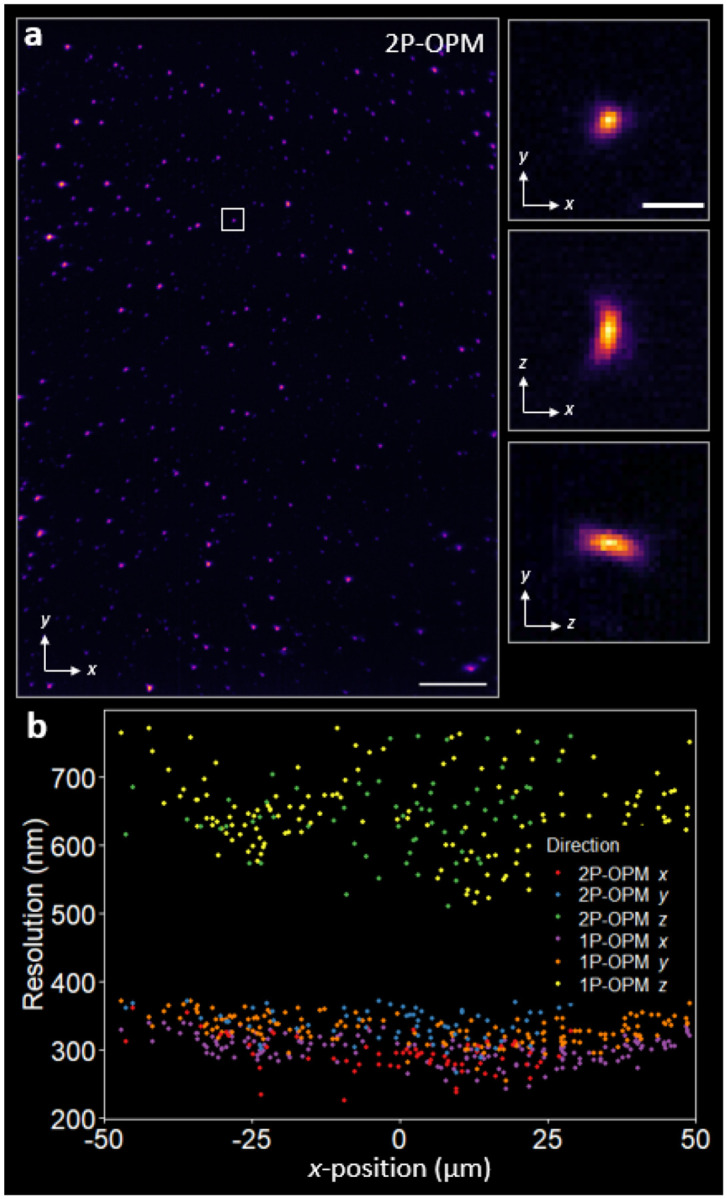
2P-OPM performance. **(a)** xy MIP of a beads field captured with 2P-OPM. Scale bar, 10μm. Insets show xy (top), xz (middle), and yz (bottom) MIPs of a representative bead from the region indicated by the white box. Zoomed-in regions of beads across an entire volume are shown in Fig. S9. Scale bar, 1μm. **(b)** x,y, and z resolution, as measured by the FWHM, across the x-field of view for 2P-OPM and 1P-OPM (N>90 beads for each mode). The mean x,y, and z FWHM ± SD values are 2P-OPM, 292 ± 40 nm, 331 ± 40 nm, 653 ± 84 nm, respectively; and 1P-OPM, 283 ± 27 nm, 324 ± 28 nm, 613 ± 60 nm, respectively.

We benchmarked the spatial resolution using 50 nm fluorescent beads immobilized on a coverslip (Methods; Fig. 2a and S10a,b). For each modality, > 90 isolated beads were analyzed from xy,xz, and yz maximum intensity projections (MIPs) via 1D Gaussian fits to line profiles. For 2P-OPM, the mean full width at half maximum (FWHM) ± SD is x=292±40nm,y=331±40nm, and z=653±84nm; for 1P-OPM, the corresponding values are x=283±27nm,y=324±28nm, and z=613±60nm (Fig. 2d and S10c,d). Because the beads sample is homogeneous and weakly scattering, and because the detection optics and illumination sheet thickness were matched between 2P and 1P (Fig. 1a,b and S7), the measured PSFs for 2P- and 1P-OPM have similar dimensions along x,y, and z.

Using off-the-shelf achromatic doublets for T3_1–3_, rather than specialized tube lenses that often trade spectral bandwidth for field, delivers uniform resolution across a $100\times 100\times 15\left(xyz\right){\;\mu \text{m}}^{3}$ volume (Fig. 2d, S9), a lateral span consistent with early high-NA OPM designs [7]. The lateral PSF is mildly anisotropic (x<y;y/x≈1.13), characteristic of high-NA OPM due to the O3 tilt, and matches our design calculations and theoretical simulations (Methods; Fig. S2 and S3). As expected, a ~0.9-μm-thick illumination plane coincident with and thinner than the detection depth of field improves axial resolution by over 30% compared to wide-field illumination. These measurements represent the highest spatial resolving power for 2P single-objective light-sheet microscopy.

### Enhanced performance in multicellular systems

**FIG. 3.**
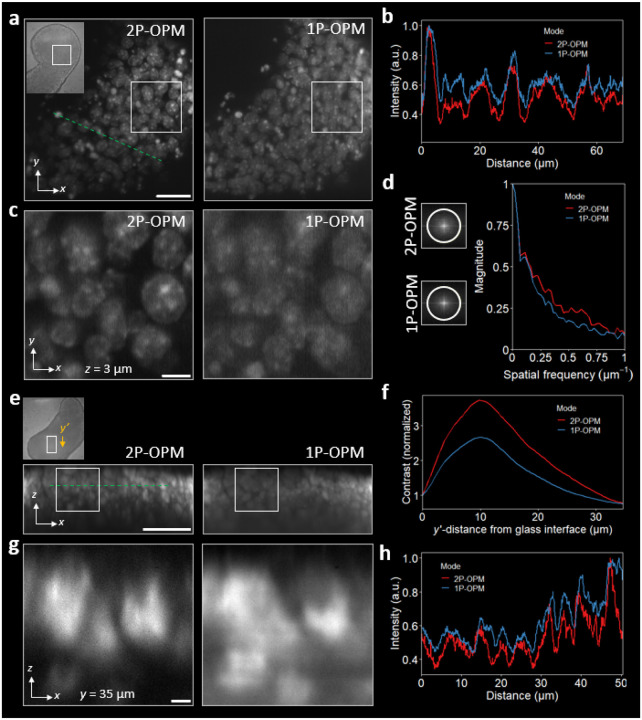
2P-OPM improves the contrast and resolution in volumetric imaging of multicellular systems. **(a)** xy MIPs of a 120-hr DAPI-stained gastruloid, captured with 2P-OPM (left) and 1P-OPM (right). Inset: transmitted light image showing the $100\times 105\times 30\left(xyz\right)\;\mu {\text{m}}^{3}$ volume imaged. Scale bar, 20μm. **(b)** Intensity profiles along the dashed green line in **(a)**, showing that 2P-OPM provides higher SNR than 1P-OPM. **(c)** Zoomed-in xy slice from the box in **(a)**, highlighting cleaner images with 2P-OPM. Scale bar, 5μm. **(d)** Fourier transforms of xy MIPs in **(a)**. Resolution bands (white circles at $1\;\mu {\text{m}}^{-1}$) highlight the increased spatial frequency content of 2P-OPM compared to 1P-OPM. The average amplitudes of the Fourier spectra (right) show that 2P-OPM reaches the noise floor more slowly, indicating superior effective resolution. **(e)** xz MIPs of a 120-hr gastruloid stained by immunofluorescence for FOXC1, recorded with 2P-OPM (left) and 1P-OPM (right). Top inset: transmitted light image showing the $70\times 100\times 30(xyz)\;{\mu \text{m}}^{3}$ volume imaged. Yellow arrow indicates the light-sheet propagation direction ($y^{\prime }$), corresponding to the $y^{\prime }$-distance from the glass interface. Scale bar, 20μm. **(f)** Quantification of image contrast as a function of $y^{\prime }$-distance from the glass interface, showing improved contrast over the full illumination depth with 2P-OPM over 1P-OPM. Each xz’ slice is normalized to the surface value. **(g)** Zoomed-in xz orthoslice from the box in **(e)**, revealing that 2P-OPM reduces background and better resolves individual cell nuclei deep in the gastruloid tissue than 1P-OPM. Scale bar, 2μm. **(h)** Intensity profiles along the dashed green line in **(e)**, demonstrating the improved SNR and optical sectioning of 2P-OPM.

Dense multicellular aggregates (such as organoids and gastruloids) present a stringent test for optical sectioning, where scattering and out-of-focus fluorescence rapidly degrade contrast under 1P excitation. The tight, planar confinement of 2P excitation mitigates these effects, reducing scattering-induced background. In line with this, 2P-OPM images of gastruloids exhibit clearer subcellular structure than 1P-OPM across matched fields of view (Fig. 3a).

Quantitative analyses reinforce this improvement. Line profiles drawn across identical trajectories (Fig. 3b) show higher SNR with 2P-OPM, reflecting the recovery of signal otherwise lost to scattering. Zoomed xy views (Fig. 3c) resolve individual cell nuclei with greater local contrast. Fourier spectra of the xy projections (Fig. 3d) show elevated mid- to high-spatial frequency content with 2P-OPM, demonstrating more faithful preservation of nuclear to subnuclear structures in the images. Together with bead-based PSF measurements in homogeneous media, these results emphasize that while detection PSFs are comparable, nonlinear excitation substantially enhances *effective* resolution in scattering tissue.

Axial projections further highlight this depth advantage. In matched xz views (Fig. 3e,g), 2P excitation suppresses out-of-focus background and maintains thin optical sections 30μm into the specimen, whereas 1P-OPM accumulates extraneous fluorescence. Intensity profiles taken along the dashed lines (Fig. 3h) exhibit narrower peaks and increased SNR, consistent with confinement of excitation to the detection focal plane. A depth-dependent contrast metric (Methods, Eq. (11); Fig. 3f), normalized at the surface, remains higher for 2P-OPM across the full 35μm illumination depth, confirming stronger sectioning in thick, densely labeled specimens. Averaged over this range, contrast is >1/3 higher for 2P-OPM than 1P-OPM. Integrated over the same range (area above unity), the contrast is ~2× larger for 2P-OPM, consistent with prior multi-objective 2P light-sheet systems [17].

### Molecular imaging under low-SNR conditions

Molecular fluorescence imaging in intact tissue is often limited by background rather than diffraction. Under linear (1P) excitation, out-of-focus fluorescence creates a near-uniform haze that raises the noise floor; the faintest signals then sit only slightly above this noise and, in practice, set the image SNR. As the background increasingly overwhelms the signal, high-fidelity photon counts decrease and shot noise hampers the effective resolution. In contrast, 2P-OPM spatially localizes excitation to the illumination and detection focal planes, keeping noise low so that dim signals remain resolvable.

**FIG. 4.**
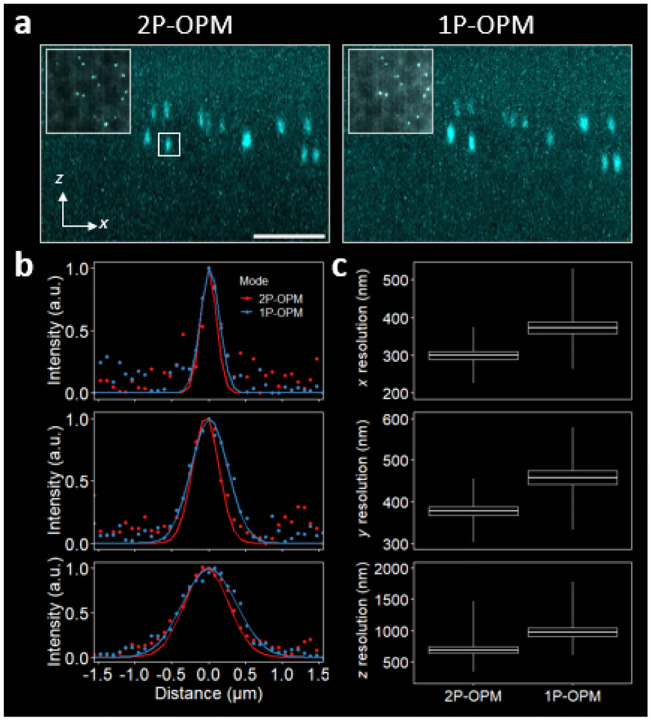
2P-OPM enhances molecular imaging performance in low SNR fluorescence. **(a)** xz MIPs of mRNA activity for the eve gene, labeled via smFISH, in a fixed *Drosophila* embryo at NC 14, comparing 2P-OPM (left) and 1P-OPM (right). Inset: xy view, highlighting activity in individual nuclei. Scale bar, 5μm. **(b)** x (top), y (middle), and z (bottom) line intensity profiles through the transcript from the subregion indicated in **(a)**, showing better resolution in all dimensions. Color points: raw data; solid lines: Gaussian fit. **(c)** x (top), y (middle), and z (bottom) resolution, measured by the FWHM; for each modality, the same (N=20) transcripts were chosen from the tissue represented in (a). All boxes denote mean ± standard error; center values are means; whiskers represent the spread of the data. The mean x,y, and z FWHM ± SD values are 2-OPM, 299 ± 46 nm, 377 ± 50 nm, 692 ± 24 nm, respectively; and 1P-OPM, 373 ± 69 nm, 458 ± 75 nm, 975 ± 30 nm, respectively.

In single-molecule fluorescence in situ hybridization (smFISH) measurements of *eve* transcripts in fixed *Drosophila* embryos, 2P-OPM yields cleaner, less anisotropic spots than 1P-OPM in identical fields (Fig. 4a). Line profiles through isolated subdiffractive transcripts (Fig. 4b) show higher SNR and narrower Gaussian fits along all axes. The mean spot FWHM ± SD improves from 373 ± 69 nm to 299 ± 46 nm in x, from 458 ± 75 nm to 377 ± 50 nm in y, and from 975 ± 30 nm to 692 ± 24 nm in z (Fig. 4c), reductions of ≈ 20%, ≈ 18%, and ≈ 29%, respectively, computed over the same N=20 transcripts. These per-axis reductions yield a ~ 2.15× recovery in 3D resolution (diffraction-limited puncta sharpened by ~53%). Because the detection PSF and light-sheet thickness are matched between the modalities, these gains reflect enhanced thin-plane illumination and background rejection via 2P, with the largest benefit along z where out-of-plane blur dominates.

### Molecular imaging *in vivo*

**FIG. 5.**
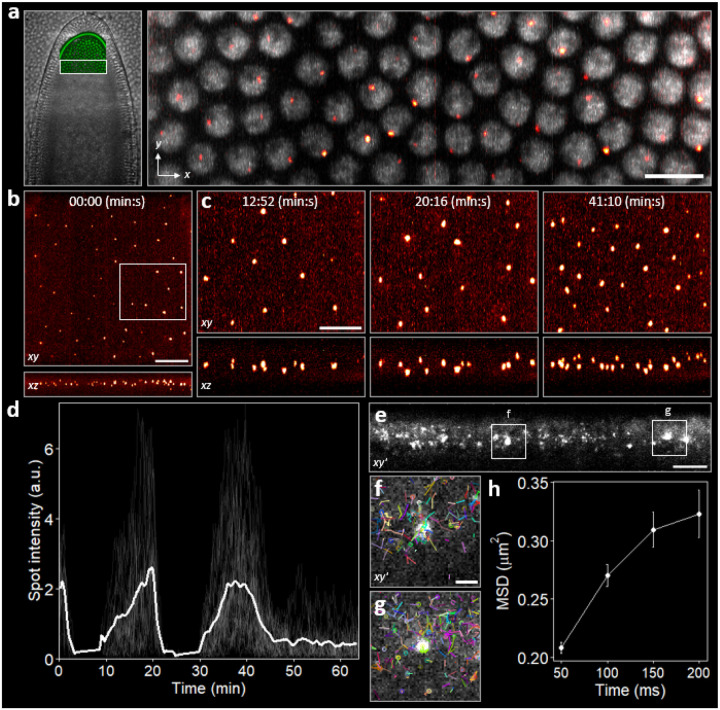
2P-OPM enables fast, multicolor, single-molecule imaging in living animals. **(a)** Left: Transmitted light (brightfield) image of a live fruit fly embryo at NC 14, expressing Bcd-eGFP (overlaid in green), marking the $100\times 115\times 15(xyz){\;\mu \text{m}}^{3}$ volume imaged with 2P-OPM. The white rectangle indicates the 3.5-μm-thick slab shown at right. Right: Zoomed xy MIP of Bcd-eGFP (gray) and nascent hb transcripts, tagged with MS2-MCP-mCherry (orange hotspots). Scale bar, 10μm. **(b)** xy (top) and xz (bottom) MIPs from a $100\times 115\times 15\;{\mu \text{m}}^{3}$ volume in a developing fruit fly embryo, showing active hb transcription loci labeled with MS2-MCP-mNeonGreen at the end of NC 12. See also Video 2. Scale bar, 20μm. **(c)** Zoomed time-lapse of NC events from **(b)**. Left: beginning of NC 13; middle: peak of NC 13; right: peak of NC 14. Scale bar, 10μm. **(d)** Transcription traces of 148hb spots sampled every ~10 s from a $100\times 15\times 15\;{\mu \text{m}}^{3}$ subvolume within a $100\times 115\times 15\;{\mu \text{m}}^{3}$ imaged field of a fly embryo. This subvolume corresponds to mid-anterior along the AP axis, centered at x/L≈0.45 (spanning ~0.37–0.53). Mean activity is overlaid (white). **(e)** Temporal standard-deviation projection showing hb mRNAs as diffraction-limited puncta and transcription sites as larger, brighter hotspots. $100\times 20\;\mu {\text{m}}^{2}$ field of view captured at 20 Hz over 50 time points (Video 3). Scale bar, 10μm. **(f,g)** Zoomed views of the boxed regions from **(e)**, showing the first time point with single-particle trajectories overlaid from the full sequence (tracks ≥2 steps; colors indicate track identity). Scale bar, 2μm. **(h)** Mean squared displacement (MSD) of hb mRNA molecules as a function of time (Δt=50-200ms). Points are mean ± standard error across step pairs; a linear fit to the first two Δt points yields an apparent ensemble diffusion coefficient of $D\approx 1.04\;\mu {\text{m}}^{2}/\text{s}$.

Having validated improved contrast and resolution in *in vitro* cell aggregates and intact fixed tissue, we next asked whether these gains translate *in vivo*. In nuclear cycle (NC) 14 embryos co-expressing Bicoid transcription factors (Bcd–eGFP) and nascent *hunchback* (hb) transcripts (MS2–MCP-mCherry), 2P-OPM enables simultaneous two-color imaging of Bcd and active transcription sites (Fig. 5a). Bcd shows nuclear-to-nuclear variability and subnuclear heterogeneity, with localized enrichments evident in nearly all nuclei, mirroring recent reports of heterogeneous Bcd distributions and transient local concentration near active transcription loci [27, 28].

Volumetric time-lapse imaging of transcriptional activity over developmental time (NC 12–14) reveals bright hb hotspots with high contrast in xy, as well as tight axial hotspots in the corresponding xz projections, representing strong optical sectioning under 2P excitation (Fig. 5b). 4D dynamics and zoomed subregions illustrate the onset and peaks of individual active transcription sites across successive nuclear cycles (Video 2 and Fig. 5c).

Our implementation of 2P light-sheet excitation delivers over an order-of-magnitude lower peak intensity at a given average power (~4.3× smaller illumination NA yields a ~ 19× smaller peak intensity), reducing the propensity for nonlinear photodamage. This headroom permits higher tolerable average power and thus higher resolution sampling in time and space. Exploiting this low-peak, high-average-power photon budget, we increased the voxel rate to 3.25 × 10^6^ s^−1^, over 5× the 6.22 × 10^5^ s^−1^ used in previous work by 2P point-scanning microscopy [13]. From a mid-anterior subvolume we then extracted 148hb transcription-site trajectories across NC 12–14 (Methods; Fig. 5d); despite >1 hr of continuous illumination with 80 mW of average power (4× higher than 2P point scanning), the fluorescence traces are not compromised, and both single-spot and mean trajectories recapitulate the expected bursty, NC dynamics observed in live embryos [13, 29]. The embryo showed no phenotypic signs of phototoxicity and progressed through normal sequences of development, as confirmed by a 16-hr bright-field time-lapse acquired after 2P-OPM imaging (Fig. S12).

Combining 2P-OPM with the MS2 stem-loop-based labeling system also allowed us to directly observe single transcripts in a living fly embryo. MS2 provides a locus-tied burst of photons from each nascent transcript, maximizing per-molecule signal, while selective-plane 2P excitation suppresses out-of-focus background and photobleaching that would otherwise contaminate single-molecule SNR and reduce localization precision under 1P excitation in a highly scattering specimen.

With a ~ 1.5× increase in average power and the 2P light sheet parked at a fixed z plane, the SNR is sufficient to resolve individual hb mRNAs as they are released from the transcription site and to track their diffusion across a $100\times 20{\mu \text{m}}^{2}$ field of view at 20 Hz (Fig. 5e and Video 3). We tracked $\sim 1.4\times 10^{3}\;hb$ molecules, comprising 8.5 × 10^3^ localizations (Fig. 5f,g). The mean detected emission was ~1.1 × 10^2^ photons per localization, with a mean precision of $\sigma_{x}=32.9\;\text{nm}$ and $\sigma_{y^{\prime }}=58.4\;\text{nm}$ (Cramer-Rao lower bound; see Methods). Single-particle tracks place the apparent ensemble diffusion coefficient of hb transcripts at $D\approx 1.04\;{\mu \text{m}}^{2}/\text{s}$ (Fig. 5h), between the fast diffusion coefficient of *Sox2* transcription factors in mammalian cells ($\sim 0.89\;\mu {\text{m}}^{2}/\text{s}$) and *Zelda* transcription factors in fly embryos ($\sim 1.55\;\mu {\text{m}}^{2}/\text{s}$), both measured by lattice light-sheet microscopy [1, 27]. This intermediate value for hb is consistent with mRNAs diffusing more slowly than freely diffusing, non-DNA-bound transcription factors in mammalian nuclei [30], and likely reflects transient interactions within the nuclear environment that slow, but do not arrest, diffusion.

### Optogenetic control and molecular readout

Optogenetics enables precise control of protein activity with light, making it a powerful tool for understanding the dynamics of cellular systems. By combining optogenetic perturbation with high-resolution imaging, one can both trigger specific subcellular events and directly visualize their spatiotemporal responses. The broad spectral excitation window of our hardware offers optimal pairing of imaging and optogenetic wavelengths. 2P activation of CRY2-based optogenetic constructs has been reported near ~850 nm and ~950 nm [31, 32], so we used 1030 nm for imaging to avoid inadvertent photoactivation. This wavelength frees the visible spectrum for optogenetic control while delivering high-contrast molecular imaging throughout the tissue.

**FIG. 6.**
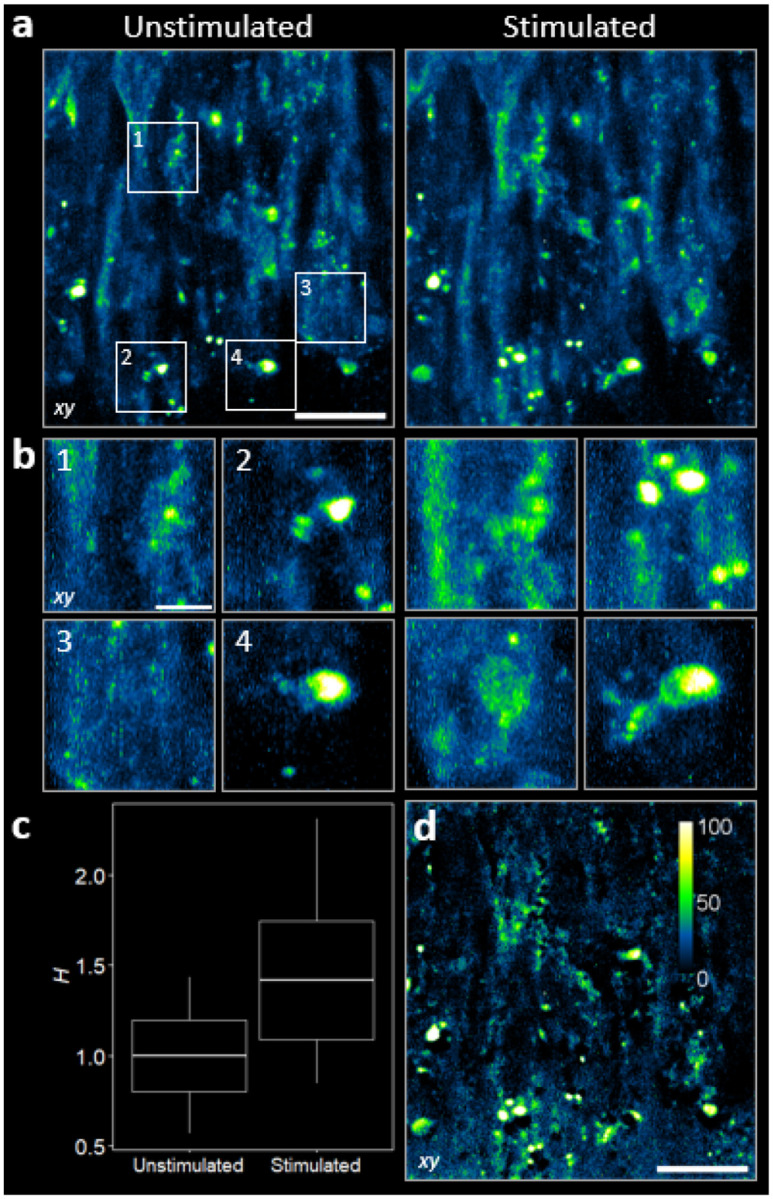
2P-OPM reveals light-controlled subcellular organization in living cells and tissues. **(a)** xy MIPs of OptoEGFR-expressing tissues before (unstimulated) and after (stimulated) LED illumination. White boxes (1–4) highlight regions where light triggers clustering and subcellular reorganization. Scale bar, 20μm. **(b)** Zoomed views from **(a)**, showing the emergence of new puncta or brightening of pre-existing ones. Scale bar, 5μm. **(c)** Heterogeneity index H measured in the ROIs from **(b)**. Light stimulation increases H by ~ 45% across all four ROI pairs. Boxes denote mean ± standard error; center values are means; whiskers represent the spread of the data. **(d)** Difference (stimulated – unstimulated) image highlighting gains after stimulation. Color map localizes the enrichment of bright puncta consistent with light-induced molecular clustering. Scale bar, 20μm.

Leveraging this spectral separation, we activated light-sensitive optogenetic epidermal growth factor receptor (OptoEGFR) with brief, wide-field blue illumination $\left(\lambda_{\text{Stim}}=455\;\text{nm}\right)$ in epithelial cells [33]. We used a 24-min stimulation train (1 s pulses every 90 s) with a time-averaged irradiance of ~1.7 mW cm^−2^, an integrated dose ~4.5–6.5× gentler than the 3 hr base regimen previously applied in [33]. 2P-OPM imaging ($\lambda_{\text{EX}}=1030\;\text{nm};\;\lambda_{\text{Em}\;\text{peak}}\approx 603\;\text{nm}$) reveals light-evoked subcellular reorganization: pre- and post-stimulation volumes show the appearance of new puncta and the brightening of pre-existing ones with high contrast throughout the tissue depth (Fig. 6a,b). Clustering was quantified by a spatial heterogeneity index H (see Methods, Eq. (21)) across four regions of interest (ROI). Under blue stimulation, H increases by ~45% across all ROI pairs (Fig. 6c); difference maps localize these gains to receptor-enriched membrane assemblies (Fig. 6d). These observations match the OptoEGFR mechanism, in which blue light triggers receptor oligomerization and activation [33]. Together with the lower stimulation dose, the spatially localized gains indicate that 2P-OPM both delivers optogenetic control and quantifies subtle, molecular-scale processes in living tissue.

## DISCUSSION

2P-OPM brings the nonlinear advantages of multiphoton excitation to high-NA single-objective light-sheet microscopy, enhancing high-resolution imaging at depth with conventional sample mounting. This reshapes the photon-budget constraints in multiphoton microscopy: By exciting an entire plane at reduced illumination NA, the photon budget is spread across many voxels, extending total illumination time per voxel and delivering over an order-of-magnitude lower peak intensity at the same average power and axial resolution to 2P point scanning. This dramatic reduction in peak intensity reduces susceptibility to higher-order nonlinear damage and allows higher tolerable average powers, enabling faster, gentler volumetric imaging than conventional 2P.

Our instrument delivers raw resolutions of ~ 300 nm laterally and ~650 nm axially, the highest reported for multiphoton OPM. In multicellular contexts, we observe ~ 2× higher contrast integrated over the full illumination depth, better transmission of spatial frequencies, and stronger optical sectioning than 1P-OPM at matched detection NA, yielding clearer cellular and subcellular images in scattering volumes. In low-SNR conditions, we find > 2× recovery in volumetric resolving power, with diffraction-limited puncta sharpened by more than 30% along the axial direction where scattering-induced blur most degrades 1P performance.

These gains and the extra bandwidth in the photon budget enable faster, more multiplexed, or more sensitive subcellular imaging *in vivo*. In living fly embryos, we can record 4D transcriptional dynamics across hundreds of individual nuclei at speeds exceeding conventional 2P point scanning by more than fivefold, simultaneously capture heterogeneous transcription-factor distributions and active gene loci, and resolve single mRNA molecules emerging from transcription sites and track their motion within the nucleus and syncytium. Beyond imaging, the same NIR excitation also permits visible optogenetic perturbation without crosstalk. OptoEGFR activation required 4.5× gentler integrated irradiance and 3× shorter stimulation than previously reported [33], suggesting that prolonged perturbation-measurement durations at high spatiotemporal resolution are possible.

Because overall 3D image quality is limited by the poorest-resolution axis, PSF anisotropy matters. As in other high-NA OPMs, lateral asymmetry from the remote-focus tilt is modest, while axial resolution is typically ~2–5× worse [7, 9, 34]. Thickening the light sheet extends propagation length and field of view but degrades z resolution and sectioning; thinning it does the opposite. A 0.5-μm-thin Bessel-like sheet approaches isotropy and triples the field of view (Fig. S5 and S7). Residual side lobes only slightly reduce SNR (Fig. S11) but redistribute power away from the main lobe, lowering effective 2P excitation (owing to the quadratic in intensity) within the detection depth of field at fixed average power, which depresses signal relative to a Gaussian sheet.

The high-NA OPM train (O1–O3, tube lenses, scan optics) also reduces NIR throughput and accumulates group-delay dispersion, broadening ultrafast pulses and diminishing 2P signal at fixed average power. Both Gaussian and propagation-invariant beams are affected, with the latter more penalized because side-lobe power lowers main-lobe intensity. Consequently, our live single-molecule mRNA imaging was confined to a fixed plane and limited to ~50 time points before molecules moved out of focus or were lost to photobleaching.

Our current 2P-OPM design leaves room for improvement. Replacing GM-x with a resonant galvo in “time-averaged” mode would illuminate each voxel multiple times within one exposure, spreading the dose in time (lower per-pass peak dose) while preserving the integrated dose per frame. This has been reported to reduce photobleaching and phototoxicity at a given average power and, when shot-noise-limited, to maintain SNR at higher sampling rates [35, 36]. High-transmission optics with visible–NIR coatings and dispersion compensation to restore bandwidth-limited pulses [37] should further boost 2P signal rates.

Even with 2P-OPM’s benefits in optically heterogeneous tissue, depth-dependent aberrations distort excitation and detection wavefronts. Adaptive optics can recover near-diffraction-limited resolution and peak signal [38–40], but adds hardware, calibration, and local, field-dependent corrections. A single correction is at best partial; tiled corrections recover resolution only by sacrificing the very speed and large-field benefits that motivate light-sheet imaging in the first place [38].

2P-OPM itself is also exacting and expensive to build and operate. The remote-focus system, two microscopes back-to-back to form a remote intermediate image with equal lateral and axial magnification [22], requires specialized optical expertise. OPM then adds a third, tilted microscope to relay and magnify an oblique plane from the remote intermediate image to the camera [5]. Room-temperature fluctuations of ~1–2°C can induce ~2-3μm axial drift in the remote-focus space [41], placing strict tolerances on O2–O3 and making drift mitigation essential to maintain long-term alignment. Ultrafast lasers for 2P excitation add cost, and demand precise co-alignment of all optical paths. Wavelength mixing can enable simultaneous 2P excitation of three fluorophores with just two synchronized beams; however, it requires tight spatiotemporal overlap and dispersion matching [42, 43]. Reducing complexity and cost will be critical for reliability, scalability, and broader access [44].

Fluorophore photophysics and photodamage thresholds ultimately bound performance. 2P cross-sections are typically smaller than for 1P, lowering signal per unit average power and limiting maximum acquisition speed; peak intensity must also stay below nonlineardamage thresholds. One-color imaging permits selection of the most photostable fluorophore, whereas multicolor often forces incorporation of less photostable labels. Continued refinement of bright, photostable 2P and NIR probes, including yellow- and red-shifted genetically encoded calcium indicators [45, 46], rhodamine-based small molecules [47], and emitters in the NIR-II window [48], will be key to faster, gentler, and highly multiplexed multiphoton imaging. Complementary triplet-state strategies, such as NIR co-illumination (to drive reverse intersystem crossing) [49] and two-step excitation through real intermediate states [50, 51], can further reduce photodamage, extend measurement duration, and accelerate frame rates at the same SNR, with two-step excitation charting a path to more compact, lower-cost systems.

These developments position 2P-OPM as an enabling technology for recording high-speed, 4D molecular movies in intact multicellular systems. Crucially, it requires no changes to standard sample mounting, substantially lowering the barrier to wider adoption. This unique combination of molecular contrast, volumetric speed, *in vivo* compatibility, and practical usability offers a quantitative bridge from fast microscopic interactions to emergent macroscopic function in living systems [52, 53]. Next-generation microscopes can incorporate structured illumination to unlock super-resolution in thick, densely fluorescent specimens [54, 55]. By relaxing resolution for field of view, whole-organoid imaging of subcellular dynamics also becomes feasible, enabling 4D single-cell tracking during self-organization [56, 57]. The same capabilities translate naturally to high-content drug screening on patient-derived organoids and 3D pathology for clinical applications [58, 59].

## METHODS

### Theory and numerical simulations

#### Detection point spread function

We generated the 2P-OPM detection PSF by considering the primary (O1), secondary (O2), and tertiary (O3) objective lenses with the model of Yang et al. [34], using code written in Python and accessible on GitHub. A plane wave passes through O2 and the pupil function is estimated after propagating out of O3. The PSF is then simulated from this pupil function. O2 (NA = 0.95) and O3 (NA 1.0) are air (n=1.0) and “solid” immersion (n=1.515) objectives, operating either in straight transmission (i.e., angle between O2 and O3 optical axes = 0°; Fig. S3a) or with O3 tilted by a 45° angle with respect to O2’s optical axis (Fig. S3b). With a 45° angle between O2 and O3, the effective pupil function of the remote-focusing system shows a compression of light in the tilted (y) direction (Fig. S3b).

To obtain FWHM measures of the theoretical resolution, the PSFs are fit to a 1D Gaussian along the three principal axes (Fig. S3). The tilted PSF is first rotated to have its principal axes along the x-,y- and z-axes and then fit with a 1D Gaussian to get the FWHM. In both the straight and tilted cases, the FWHM is further scaled by 1.515× to account for the magnification between O1 and O2, which is the ratio of the indices of refraction between O1 and O2, satisfying both the sine and Herschel condition to achieve “perfect” (i.e., aberration-free) remote refocusing [22].

#### Two-photon Bessel-like beams

We generated 2P Bessel-like excitation beams by placing a pupil-plane “layer-cake” phase mask [25, 26, 60] in a plane conjugate to the objective back pupil (Fig. S5). The mask consists of concentric glass discs; adjacent zones introduce an optical path difference δ=t(n-1) (layer thickness t, refractive index n). For ultrafast sources (pulse durations ~100–200 fs; coherence length ≲ a few tens of μm), choosing δ≫ coherence length makes the annular sub-apertures mutually incoherent. Each zone then forms its own elongated focus; their intensities add incoherently to yield an effective extended depth-of-focus excitation profile with near-Gaussian lateral width but extended axial extent.

We computed excitation foci using the vectorial Debye diffraction integral with a Fourier-domain implementation [25, 61], the same code as our previous extended depth-of-field light-sheet work [26]. A continuum of wavevectors transmitted by an objective lie on a spherical cap with a magnitude k=n/λ. Lateral and axial feature sizes map to the wavevectors along $k_{x}$ and $k_{z}$, respectively. The set of $k_{x}$ wavevectors is given by $2k\;\text{sin}\left(\alpha_{2}\right)$; the set of $k_{z}$ wavevectors is given by $k\left(\text{cos}\left(\alpha_{1}\right)-\text{cos}\left(\alpha_{2}\right)\right)$. For a conventional (central) sub-aperture using the full spherical cap $\left(\alpha_{1}=0\right)$, the axial extent is

(1) $$d_{z}=\frac{\lambda }{n\left(1-\text{cos}\left(\alpha_{2}\right)\right)}.$$

Annular sub-apertures ($\alpha_{1}\neq 0$) produce Bessel-like foci with axial extent

(2) $$d_{z}=\frac{\lambda }{n\left(\text{cos}\left(\alpha_{1}\right)-\text{cos}\left(\alpha_{2}\right)\right)}.$$

Using α=arcsin(NA/n) gives

(3) $$d_{z}=\frac{\lambda }{n\left[\text{cos}\left(\text{arcsin}\left(\frac{{\text{NA}}_{1}}{n}\right)\right)-\text{cos}\left(\text{arcsin}\left(\frac{{\text{NA}}_{2}}{n}\right)\right)\right]}.$$

Because the back pupil diameter is 2f NA (where f is the objective back focal length), Eqs. (1)–(3) convert NAs directly to the corresponding mask zone radii when properly accounting for the relay magnification between them. We determine zone sizes iteratively so each zone yields the same $d_{z}$: choose the central (low-NA) zone to set the target $d_{z}$ via Eq. (1), then pick the next annulus (NA_1_, NA_2_) from Eq. (3) so its Bessel-like focus has the same $d_{z}$, and repeat outward until the desired number of zones (or maximum NA) is reached. This construction yields the expected near-linear scaling of the depth of focus with the number of annuli.

For each zone we compute the complex electric field E(r), form the 2P excitation foci as $|E{|}^{4}$, and then sum incoherently across zones to obtain the axially elongated 2P focus. The central zone produces a Gaussian-like focus (Eq. 1); outer annuli produce Bessel-like foci (Eq. 3). The summed extended focus maintains lateral width close to that of the highest NA zone while extending the axial extent set by the design $d_{z}$ (see Fig. S5 and S6). Wavelengths and NAs closely matched the experimental parameters: $\lambda_{\text{exc}}=900\;\text{nm},{\text{NA}}_{\text{exc}}=0.5,n=1.515$, and L=4, where L is the number of annuli; this yielded a depth of focus of 10μm; the conventional high-NA Gaussian beam was identical, except with a depth of focus of 2.5μm. We experimentally validated these theoretical predictions by imaging a solution of rhodamine (Fig. S7), and characterized the performance by imaging a DAPI-stained fruit fly embryo (Fig. S11).

### Microscope optics

Refer to Fig. 1a for the beam paths and key components. Table I contains a list of acronyms and key parts used to construct the microscope; a complete parts list is in the computer-aided design (CAD) model. Fig. S1 shows a CAD and photos of the 2P-OPM instrument.

Two Ti:Sapphire lasers and an ytterbium-based laser are used for 2P excitation (red path). Intensity modulation for each ultrafast laser source is achieved using Pockels cells (PC1-PC3). The NIR beams are combined using broadband and dichroic mirrors (DM7 and DM8) and expanded to a 12-mm diameter, with an adjustable iris (I) downstream to tune the illumination NA. A layer-cake phase mask (MSK) can optionally generate a Bessel-like focus (see Methods above). CW laser sources at 405 nm (purple), 488 nm (blue), and 561 nm (yellow) are used for linear 1P excitation.

These beams are collimated and expanded by lenses L5_1–3_ and L6_1–3_to a 1.3-mm diameter and combined via broadband and dichroic mirrors (DM5 and DM6) into a single beam. This beam passes through a half-wave plate ($\lambda /2_{\text{VIS}}$) and acousto-optic tunable filter (AOTF) for modulation (blue path), then expands 7.5× via lenses L3 and L4. The illumination NA is controlled with an iris (I) before the beam is merged with the NIR light path using DM4.

Lens L2 focuses the combined visible and NIR co-linear beam onto resonant galvo (RG). The beam is then conjugated to scanning galvo mirrorrs GM-x and GM-y, and the rear focal plane of the primary objective O1 in sequence through lens L1, tube lens T2 and scan lens SL2, and SL1 and T1. For proper conjugation and positioning of the galvos, RG and GM-x are mounted on 2D translation stages; GM-y is mounted on a 3D translation stage. L2 through GM-x sit on a breadboard bolted to a y-translation stage (Fig. S1d). DM1 directs the visible and NIR light to O1; translation of the y stage offsets the beam from center so that it intersects the specimen obliquely. 3D motion-control stages translate the sample and an environmental chamber provides closed-loop control of temprature, humidity and gas concentration.

Fluorescence emission is collected through O1 and propagated through the remote-focus optics (O2 and O3) to form an image of the oblique plane within the specimen. DM2, DM3, and a broadband mirror split the fluorescence into three spectral emission channels, which are focused onto Sci-Cam1, Sci-Cam2, and Sci-Cam3 with T3_1–3_ (Fig. S4). O1–03 and Sci-Cam1–3 are all fixed on 2D translation stages for precise alignment. O3 is further mounted to a piezoelectric collar and kinematic mount (with tip/tilt) for precise positioning and alignment to the 1P and 2P light sheet (Fig. S1e).

Flip mirror FM directs the multicolor excitation beam to a transmitted wide-field illumination mode, which includes a movable dichroic mirror (MDM) and lenses L7–L8 mounted diascopically above O1. This mode also serves as a practical alignment laser for the light microscope; specifically, the correct distance between each pair of lenses in the system is confirmed with a shearing interferometer. Removing MDM permits a beam splitter (BS) to inject 455 nm and/or 625 nm LED light for bright-field illumination or photoactivation. Movable mirror MM1 diverts detected light to a wide-field inspection camera (WF-Cam) for low-magnification view-finding and macroscopic imaging (see Fig. S8 and 1), using relay lens R1 (f=100mm) and T5.

### Microscope control

All acquisitions ran on a Colfax ProEdge WX3400–class workstation equipped with an Intel Xeon W7-3445 processor operating at 2.6 GHz with 20 cores and 40 threads, 192 GB DDR5-4800 ECC RAM, and a Samsung PM9A3 3.84 TB M.2 NVMe SSD. Data display and file writing were handled in μ-Manager 2.0 [62]. A custom Python control module (“fastMC”) programmed a National Instruments X-Series DAQ (NI-PCIe-6321 with NI-BNC-2110 breakout) via the nidaqmx API to generate synchronized hardware triggers (TTL pulses) and analog waveforms that control four cameras (Sci-Cam1–3, plus WF-Cam), the y-scan galvo (GM-y), the lasers (AOTF/Pockels), and the bright-field/optogenetic LEDs. All DAQ tasks (counters, digital lines, analog outputs) were precomputed and hardware-clocked for the acquisition duration within device limits. fastMC incorporates and adapts elements from the acquisition scripts of [34].

Sci-Cam1–3 were run in “Exposure Control” (hardware) and rolling shutter mode, in which each TTL pulse defines both the start and the duration of an exposure (pre-defined settings are ignored). fastMC uses a primary counter to: mark frames (2D) or y-stacks (3D), and start the GM-y (if 3D imaging) and the LED (both driven by pre-determined signal trains). A gated secondary counter emits the exposure-controlling pulse train accepted by Sci-Cam1–3. The exposure pulse has duty cycle ~0.98 and a period equal to the requested exposure, which ensures trigger acceptance across ROIs/readout heights and enables maximal full-sensor rates (e.g., 100 fps). See Fig. S13 for the timing diagram and the schematic of device-DAQ connections.

GM-y was driven from a ±5 V waveform (stepped once per frame during volumetric imaging) so the sample remained effectively stationary during each rolling-shutter exposure. Laser power was controlled by an analog output to the AOTF/Pockels with a concurrent TTL blanking line automatically emitted by any one of Sci-Cam1–3; excitation was on only during exposure windows and off during readout, inter-frame gaps, and galvo retrace. LED illumination offers two modes: a stacksynchronized “fraction” mode (LED on for a chosen fraction of each y-stack) and an independent “timed” mode (a user-specified on window starting with acquisition).

All DAQ tasks (counters, digital lines, analog outputs) were precomputed and hardware-clocked for the acquisition duration within device limits. fastMC was used specifically for high-speed 2D/3D acquisitions requiring deterministic hardware timing and sustained multi-camera recording without drops; routine preview and slower measurements were run directly in μ-Manager. Multi-dimensional data were saved by μ-Manager (NDTiff/TIFF series) directly to a network-attached storage (NAS) server (Synology DS1821+ 8-Bay Diskstation) connected via 10 GBe Ethernet to the acqusition PC.

### Sample preparation

#### Cleaning and sterilization of coverslips

Glass coverslips (#0; Thorlabs, CG00K) were cleaned following an alkaline–peroxide protocol [54]. A fresh 150 mL solution of hydrogen peroxide (H_2_O_2_, 50%), ammonium hydroxide (NH_4_OH, 30%), and H_2_O was prepared at a 1:1:5 volume ratio. Coverslips were immersed and soaked for 12 hr at room temperature, then rinsed thoroughly in deionized water (5×) and air-dried in a dust-free container. Immediately before use, coverslips were sterilized by a brief methanol rinse followed by flaming and allowed to cool completely.

#### Fluorescent beads

Clean 5–8 mm^2^ rectangular coverslips were coated with 0.1 mg mL^−1^ poly-D-lysine (30μL per coverslip), air-dried for 15 min, and rinsed in water. Sub-diffractive fluorescent beads (50 nm diameter; Dragon Green, Bangs Laboratories, FSDG001) were diluted ~10^6^× in ethanol; 30μL was deposited on the coated coverslip and allowed to evaporate and dry. A small drop of immersion oil (Olympus) was applied to immobilize and index-match the dried beads for PSF measurements. Samples were stored in the dark at room temperature and reused until bleached.

#### mESC culture

Mouse embryonic stem cells (mESCs) 129/SvEv (EmbryoMax) were cultured in 0.1% gelatin-coated sixwell plates in a humidified incubator (5% CO_2_, 37 °C). Cells were maintained in basal growth medium comprising Glasgow Minimum Essential Medium (G-MEM, 11710-035) supplemented with 10% ESC qualified fetal bovine serum (R&D Systems, S10250), 1:100 GlutaMAX (Gibco, 35050–061), 1:100 MEM non-essential amino acids (Gibco, 11140-050), 1 mM sodium pyruvate (Gibco, 11360–070), 100μM 2-mercaptoethanol (Gibco, 21985-023), and 1% penicillin-streptomycin (10,000 units/mL, Gibco, 15140-122). The basal growth medium was additionally supplemented with 1000 units/mL LIF (Millipore Sigma, ESG1107), 2μM PD 0325901 (Tocris, 4192), and 3μM CHIR 99021 (Tocris, 4423) to maintain pluripotency. The medium was replaced every day and cells were passaged every other day using Trypsin (Gibco, 12605-010) dissociation and a seeding density of 3×10^5^ cells per well.

#### Gastruloid culture

mESC-derived gastruloids were generated as described in [63]. In-house N2B27 medium was freshly prepared maximally one week in advance of the experiments as follows: 1:1 mix of DMEM/F12 +GlutaMax (Gibco, 10565-018) and Neurobasal (Gibco, 21103-049), supplemented with 100μM 2-mercaptoethanol, 1:100 N2 (Gibco, 17502–048), 1:50 B27 (Gibco, 17504-044) and 1% penicillin-streptomycin (10,000 units/mL). Cell seeding for gastruloid generation was performed manually using a multipipette and the cell counts were determined with an automatic cell counter (Countess 3 Automated Cell Counter, Invitrogen). For cell dissociation from adherent mESC cultures, cells were treated with Trypsin and immediately resuspended in N2B27 medium. The dissociated cells were seeded into Costar Low Binding 96-well plates (Costar, Corning, 7007) with 300 cells per 40μL per well. Gastruloids were left untouched for 48 hrs to facilitate aggregation. At 48 hrs, the resulting spheroids were subjected to a 24-hr pulse of Wnt agonist through the addition of 150μL of 3μM CHIR 99021 (Chiron) in N2B27 to each well. Subsequently, 150μL of the medium was replaced every 24 hrs until gastruloid collection at 96 or 120 hrs.

#### Immunofluorescent staining of gastruloids

Gastruloids were collected from the well plate, pooled in a 15 mL falcon tube, and washed once with PBS with Mg2+ and Ca2+ (PBS++, Gibco, 14040-133). After gastruloids sank to the bottom of the tube, PBS++ was removed and gastruloids were subsequently fixed in 10 mL 4% paraformaldehyde solution (PFA, Thermo Scientific Chemicals, 30525-89-4) for 2 hr at 4 °C. Afterwards, gastruloids were washed twice with 10 mL PBSF (10% FBS in PBS++) for 15 min at room temperature, resuspended in 1 mL in PBS++ and stored at 4 °C (for several weeks). For the immunofluorescent staining, gastruloids were permeabilized in 10 mL PBSFT (10% FBS and 0.03% Triton in PBS++) and incubated for 2 hrs at room temperature. Gastruloids were then incubated in 0.5 mL PBSFT containing 4’,6-diamidino-2-phenylindole (DAPI) and primary antibody overnight at 4 °C (see Table II for details on antibodies and concentrations). The next day, gastruloids were washed three times with 10 mL PBSFT for 30 min each at room temperature and subsequently incubated in 0.5 mL PBSFT containing secondary antibody and DAPI overnight at 4 °C (Table II). Gastruloids were subsequently washed twice in 10 ml PBSFT and once in PBS++ at room temperture for 30 min each. All incubations and washes were performed under nutation. For gastruloid mounting, all excess PBS++ was removed from the tube and replaced with 200μL mounting medium composed of 50:50 Aqua-Poly Mount (Polysciences, 18606-20) and PBS++. Gastruloids in mounting medium were then transferred to a round 35-mm glass-bottom dish (No. 0 coverglass, MatTek Corporation, P35G-0-14-C), covered with a coverglass, and sealed with nail polish.

#### smFISH probe synthesis

Probes were designed computationally with a 25-nucleotide hybridization region with a melting temperature of at least 45°C, and specific 20-nucleotide forward primer and 30-nucleotide reverse primer with an incorporated T7 promoter. Probe synthesis followed a similar protocol outlined in [64]. Briefly, probes were ordered using an oligo library from IDT at 10pmol/oligo concentration. Probes were amplified with PCR and then treated with a HiScribe T7 (E2050) in-vitro-transcription (IVT) utilizing the incorporated T7 promoter. A specific ATTO-conjugated primer (also from IDT) was added and incorporated into the probes following a reverse transcription reaction with the Maxima H Minus RT (K1652) kit. Resulting ssDNA probes were treated with RNAse A, clean, and stored at 4°C.

#### smFISH on fly embryos

Oregon–R (Ore-R) fly stocks were maintained on standard cornmeal medium. Embryos were fixed in 4% formaldehyde and stored in methanol at −20°C. smFISH targeted the exonic region of the *even-skipped* gene (*eve*) using ATTO 565- and ATTO 633-conjugated probes. Hybridization was performed with 750 ng of conjugated probe in 5× SSC buffer (Thermo Fisher Scientific, 15557044), 50% formaldehyde, and 0.1% Tween-20 at 37°C overnight. Embryos were washed, mounted in ProLong Gold (Invitrogen), and allowed to cure overnight prior to imaging.

#### Genetic crosses for live-fly imaging

To obtain embryos co-expressing Bcd and nascent hb (Fig. 5a), female virgins expressing Bcd–eGFP [65] were crossed to males carrying a maternal MS2 coat protein (MCP) line (*nanos*>NLS–MCP–mCherry). Female offspring (*bcd–eGFP/+; nanos>NLS–MCP–mCherry/+; +/+*) were then crossed to males homozygous for a hb–MS2 reporter in which the hb promoter drives a transcript containing 48 MS2 stem–loop repeats followed by the *lacZ* coding sequence. The 48× cassette was generated by duplicating a 24× MS2 cassette from piB-hbP2PP2E-24MS2-lacZ-tub(3’UTR) via NcoI and AvrII excision and insertion into the same vector digested with NcoI and SpeI (SpeI site upstream of the original cassette), resulting in tandem 48× MS2. The line was integrated by BestGene, Inc. into BDSC #27388, genotyped, and transcription preliminarily confirmed by confocal microscopy. Embryos from this cross were collected and used for live imaging.

For Fig. 5b–e, female virgins of a line carrying nuclear markers and MCP (*yw; His2Av–mRFP; nanos>NLS–MCP–mNeonGreen*) [29, 66] were crossed to wildtype (Oregon–R) males to reduce background fluorescence. Female offspring (*yw/+; His2Av–mRFP/+; nanos>NLS–MCP–mNeonGreen/+*) were then crossed to males expressing the same hb–MS2 reporter as above (48× MS2–*lacZ*). Embryos from this cross were collected and used for live imaging.

#### Fly mounting for live imaging

Fly embryo preparation for live imaging was adapted from previous work [13, 29]. A ~ 2 × 2 cm^2^ air-permeable membrane was coated with heptane glue, made by dissolving double-sided Scotch tape in heptane. The membrane was left to dry for ~10 min while embryos were collected, leaving an adhesive surface. Flies were caged on agar plates for 2 to 2.5 hrs, and the laid embryos were transferred to a piece of double-sided tape with a dissection needle, hand-dechorionated, and placed on the glued membrane, with the dorsal side up (i.e., facing the objective lens). After mounting, we immersed embryos in Halocarbon 27 oil (Sigma) and sandwiched the specimen between the membrane and a clean coverslip (see Cleaning and sterilization of coverslips section above). Excess oil is removed via lens tissue wipes (Thorlabs, MC-5) as needed.

#### OptoEGFR cell culture

RPE-1 cells expressing the OptoEGFR system were used from Suh, Thornton *et al*. [33]. OptoEGFR was introduced by lentiviral transduction of a construct encoding an N-terminal membrane localization tag fused to the OptoDroplet light-inducible clustering system (FUS^N^–FusionRed–Cry2) and the cytosolic domains of EGFR, as described in [33]. Cells were cultured in DMEM/F12 (Gibco, 11320-033) supplemented with 10% fetal bovine serum (R&D Systems, 26140079), 1% L-glutamine (Gibco, 25030-081), and 1% penicillin-streptomycin (10,000 units/mL; Gibco, 15140-122). All cells were maintained at 37 °C and 5% CO_2_ in a humidified incubator. Cells were tested to confirm the absence of mycoplasma contamination.

For imaging, glass-bottom plates (No. 1.5 coverglass, CellvIs, P96-1.5H-N) were coated with $10\;\mu \text{g}{\;\text{mL}}^{-1}$ fibronectin dissolved in PBS at 37 °C for a minimum of 30 min. Cells were then seeded on glass-bottom 96-well plates at ~4×10^4^ cells per well 24 hrs before imaging. To enhance adhesion, 100μL of cell suspension was first added per well and briefly centrifuged (30 s); after adhesion, an additional 100μL of full medium was added. Three hrs before imaging, the growth medium was replaced with serum-free starvation/imaging medium (DMEM/F12 with 1% L-glutamine and 1% penicillin–streptomycin; no FBS).

### Imaging conditions

Detailed imaging conditions for each experiment can be found in Table SI.

### Image processing and analysis

#### Image transformation and reconstruction

Both 2P- and 1P-OPM acquire volumes in an oblique (non-Cartesian) coordinate system (Fig. 1c,d), so we transform stacks to conventional microscope coordinates for most visualization and analysis (Fig. S14a–c). The scan axis is tilted (by 45°) relative to the image plane; successive frames are therefore displaced laterally and axially, yielding a sheared stack. We correct this by an affine transformation that deskews, rescales, and rotates the volume into conventional microscope coordinates (Fig. S14c). We calculate a new coordinate vector for every single voxel within a volume, so reconstruction can be computationally expensive, especially with time-series and/or multicolor data. To accelerate our reconstruction pipeline, we load our data into a Python script with GPU acceleration (CuPy), which speeds processing by an order of magnitude over CPU-only code.

In conventional z-stacking, a 3D volume is assembled from successive 2D frames separated by an experimentally known z step; specifying the inter-frame spacing relative to the in-plane (sensor) pixel size a suffices to render voxels at the correct scale (Fig. S14a). In OPM, by contrast, the scan direction is effectively oblique, so each frame advances in $z^{\prime }$ and is laterally offset in $y^{\prime }$ (Fig. S14b); here x and $y^{\prime }$ denote the in-plane (camera) axes and $z^{\prime }$ the axial direction along the detection tilt. We account for both components of the scan step during deskewing/rotation (Eqs. 4–7) to obtain a reconstructed volume in conventional coordinates (Fig. S14c).

Let $y_{\text{step}}$ be the physical scan increment per frame and θ the light-sheet angle (Fig. 1c and S14b). The per-frame displacements along the oblique axes are

(4) $$d_{\text{skew}}=y_{\text{step}}\;\text{cos}\;\theta ,$$

(5) $$d_{\text{scale}}=y_{\text{step}}\;\text{sin}\;\theta .$$

Expressed in pixel units using the in-plane pixel size a(μm/pixel),

(6) $$s_{k}=\frac{d_{\text{skew}}}{a},$$

(7) $$s_{c}=\frac{d_{\text{scale}}}{a}.$$

We apply three affine operations to homogeneous coordinates ${\left[\begin{array}{llll} x & y & z & 1 \end{array}\right]}^{⊤},\text{Skew}(K)$, Scale(S), then Rotation(R), in that (non-commutative) order (Fig. S14c):

(8) $$\left[\begin{array}{l} x\\ y\\ z\\ 1 \end{array}\right]=R\left(\theta \right)\underset{S\cdot K}{\underbrace{\left[\begin{array}{cccc} 1 & 0 & 0 & 0\\ 0 & 1 & s_{k} & 0\\ 0 & 0 & s_{c} & 0\\ 0 & 0 & 0 & 1 \end{array}\right]}}\left[\begin{array}{c} x^{\prime }\\ y^{\prime }\\ z^{\prime }\\ 1 \end{array}\right].$$

The final rotation maps the oblique stack back to the conventional frame:

(9) $$R(\theta )=\left[\begin{array}{cccc} 1 & 0 & 0 & 0\\ 0 & \text{cos}\;\theta & -\text{sin}\;\theta & 0\\ 0 & \text{sin}\;\theta & \text{cos}\;\theta & 0\\ 0 & 0 & 0 & 1 \end{array}\right].$$

(Pre- and post-translation matrices recenter the volume so rotations act about its origin; omitted here for clarity.) After applying the affine transform, we resample by trilinear interpolation onto a regular Cartesian grid, yielding isotropic voxels (a×a×a); this deskewed and rotated volume is the reconstructed output (Fig. S14c, right).

#### PSF benchmarking

Beads were detected and fit in PSFj to extract ${\text{FWHM}}_{\text{min}},{\text{FWHM}}_{\text{max}},{\text{FWHM}}_{z}$, and centroids [67]. Lateral principal axes were mapped to microscope axes by assigning the shorter in-plane width to x and the longer to y (parallel to the remote-refocus tilt). Analysis covered the full field of view; we retained unsaturated, isolated beads meeting PSFj’s fit-quality criterion ($R^{2}\geq 0.9$) and report mean ± SD and N.

#### Image contrast

We quantified image contrast in Fig. 3f following prior work [2, 35, 68]. For an image with N pixels and intensities $I_{j}$, the mean and standard deviation are

(10) $$\overset{‾}{I}=\frac{1}{N}\sum_{j=1}^{N}I_{j},\;\sigma_{I}=\sqrt{\frac{1}{N-1}\sum_{j=1}^{N}{\left(I_{j}-\overset{‾}{I}\right)}^{2}}.$$

We define contrast as the standard deviation divided by the mean (the coefficient of variation),

(11) $$\text{Contrast}=\frac{\sigma_{I}}{\overset{‾}{I}}.$$

We computed a depth-dependent contrast profile $\text{Contrast}\left(y^{\prime }\right)$ from individual $xz^{\prime }$ orthoslices as a function of $y^{\prime }$ (distance from the glass interface) across the full illumination depth, and normalized it to the surface value, so that Contrast(0) = 1 for each modality.

The depth-averaged contrast is

(12) $$\bar{\text{Contrast}}=\frac{1}{L}\int_{0}^{L}\text{Contrast}\left(y^{\prime }\right)dy^{\prime },$$

where L denotes the analyzed depth span. We evaluate $\bar{\text{Contrast}}$ as the discrete mean of the sampled $\text{Contrast}\left(y^{\prime }\right)$ values over the profile.

The depth-integrated contrast is

(13) $${\text{Contrast}}_{\text{int}}=\int_{0}^{L}\text{max}\left\{\text{Contrast}\left(y^{\prime }\right)-1,0\right\}dy^{\prime },$$

computed from the sampled profile using the standard trapezoidal sum. Reported improvements are ratios of ${\text{Contrast}}_{\text{int}}$ (or $\bar{\text{Contrast}}$) for 2P-OPM relative to 1P-OPM under matched detection optics.

#### Transcription-site spot tracking and signal integration

Transcription spot segmentation, tracking, and quantification were performed in Imaris (Oxford Instruments) using Spot segmentation. Spot detection used background subtraction with estimated diameters of 1μm in xy and 1.25μm in z. Candidate spots were filtered by Quality and Intensity Sum (> 2230) to retain low-SNR transcription puncta while suppressing isolated noise.

Tracks were computed with the Brownian motion model (max distance = 2.36μm, max gap size = 3; no gap filling). Short tracks were excluded using Filter Tracks: Track Duration.

For each accepted track and time point, signal was quantified as the Intensity Sum inside a fixed, ellipsoidal ROI matching the detection diameters. Local background was the mean intensity in a concentric shell with outer diameters 2× the ROI (shell = outer minus inner). The resulting background-subtracted traces were taken directly (no temporal gap filling).

#### Single-molecule detection and tracking

Single molecules were detected and tracked in TrackMate 7.12.2 (Fiji) [69, 70]. Detection used the Difference-of-Gaussian (DoG; LoG approximation) detector with an estimated object diameter = 0.347μm, quality threshold = 3, sub-pixel localization enabled, and no median prefiltering.

Tracks were built with a linking max distance = 1μm, gap-closing max distance = 1μm, and gap-closing max frame gap = 1 (simple LAP tracker; non-branching; gap closing only).

#### Photon calibration and localization precision

Spot intensities (digital numbers, DN) from TrackMate were converted to detected photons using the camera’s conversion gain $g\left(e^{-}/\text{DN}\right)$ and quantum efficiency η. We measured an electronic offset O (DN) by averaging over 100 dark images acquired with the same exposure and no illumination. For each localization, the detected signal photons and the residual background per pixel (photons/pixel) are

(14) $$N_{\gamma }=\frac{g}{\eta }\left[\left(I_{\text{spot}}-O\right)-\left(I_{\text{bg}}-O\right)\right],\;b_{\gamma }=\frac{g}{\eta }\left(I_{\text{bg}/\text{pix}}-O\right),$$

where $I_{\text{spot}}$ is the integrated spot intensity (DN) from the DoG fit, and $I_{\text{bg}}$ and $I_{\text{bg}/\text{pix}}$ are the local background (DN) estimated around the spot (total within the spot ROI and per pixel, respectively; $I_{\text{bg}}=N_{\text{mask}}I_{\text{bg}/\text{pix}}$ with $N_{\text{mask}}$ the number of pixels in the spot integration mask).

Localization precision was estimated using the Cramér-Rao lower bound for a 2D Gaussian PSF following Thompson *et al*. [71] and the closed-form refinements of Mortensen *et al*. [72]. With pixel size a (in μm), PSF standard deviations in camera coordinates $\left(x,y^{\prime }\right)$ denoted $\sigma_{\text{PSF}x}$ and $\sigma_{\text{PSF}y^{\prime }}$ (in μm), detected photons per localization $N_{\gamma }$, and residual background per pixel $b_{\gamma }$ (photons/pixel), the per-localization variances are

(15) $$\sigma_{\text{loc},x}^{2}=\frac{\sigma_{\text{PSF}x}^{2}+a^{2}/12}{N_{\gamma }}+\frac{8\pi \;\sigma_{\text{PSF}x}^{4}\;b_{\gamma }}{a^{2}N_{\gamma }^{2}},\;\sigma_{\text{loc},y^{\prime }}^{2}=\frac{\sigma_{\text{PSF}y^{\prime }}^{2}+a^{2}/12}{N_{\gamma }}+\frac{8\pi \;\sigma_{\text{PSF}y^{\prime }}^{4}\;b_{\gamma }}{a^{2}N_{\gamma }^{2}}.$$

PSF widths were obtained from smFISH puncta measured in the fly embryo (Fig. 4), to recaptiulate the scattering and aberrations present at depth in tissue. Because the detection plane is tilted by $\theta =45^{\circ }$ in the yz-plane (i.e., about x; see Fig. 1c and S2), the $y^{\prime }$ PSF mixes high-resolution, in-plane lateral blur with broader axial blur, degrading localization precision along $y^{\prime }$ relative to x for otherwise identical photon/background conditions. To account for this, we used the value $\sigma_{\text{PSF}y^{\prime }}=\sqrt{\sigma_{y}^{2}{\text{cos}}^{2}\theta +\sigma_{z}^{2}{\text{sin}}^{2}\;\theta }$, while $\sigma_{\text{PSF}x}$ is simply the x width.

Using Eq. (15), we computed a per-spot localization precision from each spot’s own $N_{\gamma }$ and $b_{\gamma }$ and then averaged across all localizations:

(16) $$\sigma_{x}=\left\langle \sigma_{\text{loc},x}\right\rangle ,\;\sigma_{y^{\prime }}=\left\langle \sigma_{\text{loc},y^{\prime }}\right\rangle .$$

#### Ensemble diffusion coefficient

Tracks were analyzed at a time interval Δt=50ms (20 Hz). For each consecutive time step we computed $\left(\Delta x,\Delta y^{\prime }\right)$ and the squared displacement $\Delta r^{2}=(\Delta x{)}^{2}+{\left(\Delta y^{\prime }\right)}^{2}$. Averaging over all consecutive steps gives

(17) $${\text{MSD}}_{\Delta t}=\left\langle \Delta r^{2}\right\rangle ,\;{\text{MSD}}_{\Delta t}^{\text{corr}}={\text{MSD}}_{\Delta t}-2\left(\sigma_{x}^{2}+\sigma_{y^{\prime }}^{2}\right),$$

which removes localization variance along x and $y^{\prime }$. Assuming 2D free diffusion at short intervals,

(18) $$D=\frac{{\text{MSD}}_{\Delta t}^{\text{corr}}}{4\Delta t}.$$

Uncertainty on ${\text{MSD}}_{\Delta t}$ is the standard error across consecutive-step squared displacements; uncertainty on D follows from Eq. (18).

#### MSD versus time interval

For Fig. 5h, we extended the analysis to time intervals τ=LΔt with L=1,2,3,4. Within each track, positions separated by τ were paired to compute

(19) $$\text{MSD}\left(\tau \right)=\left\langle [x(t+\tau )-x(t){]}^{2}+{\left[y^{\prime }\left(t+\tau \right)-y^{\prime }\left(t\right)\right]}^{2}\right\rangle -2\left(\sigma_{x}^{2}+\sigma_{y^{\prime }}^{2}\right),$$

averaged over all valid pairs at that τ across tracks. Points show mean ± standard error; we display the first four intervals (50–200 ms) to avoid bias from dwindling statistics at longer τ. At short intervals the MSD grows linearly with Δt; a fit to the first two intervals yields a slope ≈4D, as expected for free diffusion.

#### Heterogeneity index

For each boxed xy MIP in Fig. 6b with pixel intensities $I_{j}$, let $\overset{‾}{I}$ and $\sigma_{I}$ denote the mean and standard deviation as defined above (Eq. (10)–(11)). Defining the local dynamic range

(20) $$R=I_{\text{max}}-I_{\text{min}}.$$

We quantify clustering by a dimensionless heterogeneity index

(21) $$H=\frac{\sigma_{I}^{2}}{R^{2}}.$$

H was computed per xy MIP for stimulated and unstimulated conditions. H is invariant to global multiplicative scaling and specifically reflects changes in spatial unevenness (more/larger/brighter puncta) rather than overall brightness. In contrast, raw variance $\sigma_{I}^{2}$ increases with global gain and can overstate clustering when images are uniformly brighter.

#### Visualization and Software

Figures and videos were made with FIJI [70], Imaris (Oxford Instruments), napari [73], R [74], and MATLAB R2020a-R2024a (MathWorks) software.

## Supplementary Material

1

## Figures and Tables

**TABLE I.** Key optical components and acronyms used in Fig. 1.

Acronym	Part/description (vendor, model)
O1	Primary objective (Olympus 60×, NA 1.49, oil immersion)
O2	Secondary objective (Olympus 40×, NA 0.95, air)
O3	Tertiary objective (Applied Scientific Instrumentation 40×, NA 1.0, glass)
T1–T3	Tube lens with labeled focal length (Edmund Optics 49–287)
T5	Tube lens (Thorlabs AC254-050-A-ML)
SL1–SL2	Scan lens (Plössl; composed of two f=200mm VIS-NIR achromatic doublets; Edmund Optics 49–392)
GM-y	y galvo for OPM galvo scanning/descanning (Thorlabs GVS011)
MM1	Movable mirror 1
RL1	Relay lens 1 (f=100mm)
WF-Cam	View-finding camera for wide-field imaging through O1 (Basler ace acA2040-55um)
DM1	Dichroic mirror 1 (Semrock Di01-R405/488/561/635/800-t1-25×36)
DM2	Dichroic mirror 2 (Semrock Di03-R488-t3-50.8×55)
DM3	Dichroic mirror 3 (Semrock Di03-R561-t3-50.8×55)
Sci-Cam1–3	PCO edge4.2 Camera Link HS
L1–10	Lenses with labeled focal lengths (includes diascopic L7–L8)
GM-x	x galvo for scanning beam into sheet
RG	Resonant galvo (sheet wobble in $xy^{\prime }$ to reduce shadowing; Cambridge Technology CRS04)
DM4	Dichroic mirror 4 (Semrock FF665-Di02-25×36)
FM	Flip mirror
I	Iris, adjustable (illumination NA control)
AOTF	Acousto-optic tunable filter (continuous-wave laser modulation; AA Opto-Electronic AOTFnC-400.650-CPCh-TN; 400–650 nm, 2.5 mm aperture)
DM5	Dichroic mirror 5 (Semrock FF665-Di02-25×36)
DM6	Dichroic mirror 6 (Semrock FF665-Di02-25×36)
MDM	Movable dichroic mirror (diascopic module above O1)
BS	Beamsplitter (LED injection for bright-field)
DM7	Dichroic mirror 7 (Semrock FF850-Di01-25×36)
DM8	Dichroic mirror 8 (Semrock FF980-Di01-25×36)
PC1–PC3	Pockels cells (ultrafast laser modulation; ConOptics M350-80-02 &M302RM)
λ/2	Half-wave plate, where the subscripts VIS and NIR refer to the visible and near-infrared wavelengths
MSK	Layer-cake phase mask (Bessel-like excitation; LightMachinery, custom; see Fig. S6)
Ti:Sapph	Ti:Sapphire lasers (Coherent Chameleon Ultra I; 680–1040 nm, 2.9 W @800 nm, 80 MHz rep rate, 140 fs pulse duration)
Yb	Ytterbium-based laser (Light Conversion, FLINT-7W-SH; 1030 nm, 7 W, 11.4 MHz rep rate, sub-120 fs pulse duration)
405 nm	405 nm laser (Coherent OBIS LX 1185050, 200 mW)
488 nm	488 nm laser (Coherent Sapphire LP, 300 mW)
561 nm	561 nm laser (Coherent Sapphire LP, 300 mW)
455	455 nm LED for bright-field and/or optogenetic stimulation (Thorlabs M455L4; 1445 mW)
625	625 nm LED for bright-field and/or optogenetic stimulation (Thorlabs M625L4; 920 mW)

**TABLE II.** Primary and secondary antibodies and fluorescent dyes.

Antibody	Species	Catalog	Vendor	Dilution
BRA	Goat	AF2085	R&D Systems	1:100
FOXC1	Rabbit	EPR20678	Abcam	1:500
SOX2	Rat	14-9811-82	eBioscience	1:200
anti-goat AF-488	Donkey	A-11055	Invitrogen	1:500
anti-rabbit AF-546	Donkey	A-11010	Invitrogen	1:500
anti-rat AF-647	Donkey	A78947	Invitrogen	1:500
DAPI	—	D3571	Invitrogen	1:500

## Data Availability

The opto-mechanical computer-aided design (CAD) model and associated parts are available at github.com/kdizon/2P-OPM/CAD. Representative image data are available at github.com/kdizon/2P-OPM/data. Associated videos (Video 1–3) are available at github.com/kdizon/2P-OPM/movies.

## CODE AVAILABILITY

Python code for instrument control/acquisition (FastMC) and volume reconstruction are available at github.com/kdizon/2P-OPM/code.
