# Supplementary material for: Depth-enhanced molecular imaging with two-photon oblique plane microscopy
Source: ArXiv. 2025 Nov 12:arXiv:2511.09462v1. Preprint. [Version 1] (PMC12642766)
Supplement: 1 [file NIHPP2511.09462V1-supplement-1.pdf]

SUPPLEMENTARY INFORMATION

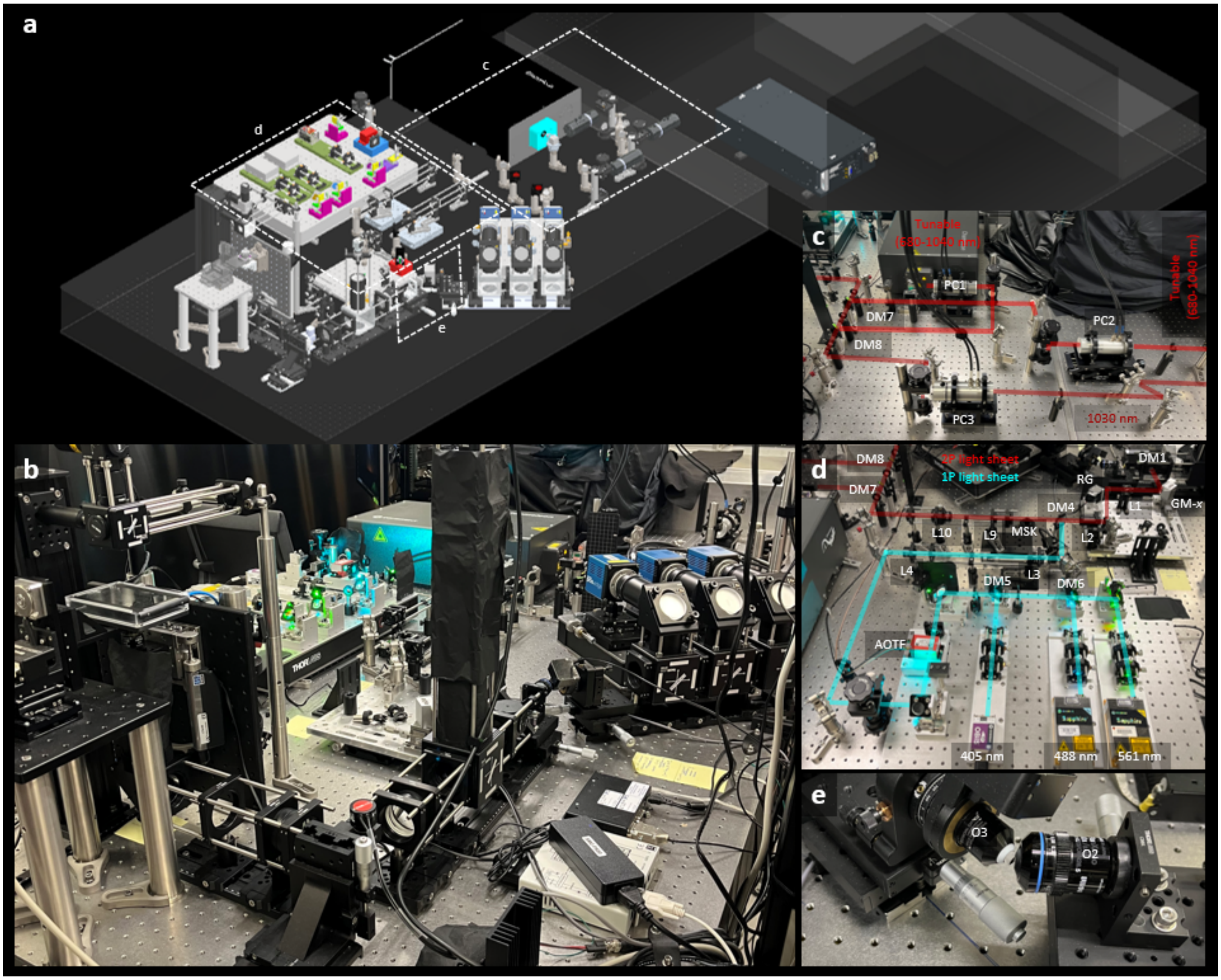

FIG. S1. **Opto-mechanical design and implementation of the two-photon oblique plane microscope.**
(a) 3D opto-mechanical solid model of 2P-OPM. Complete details of the optical system are given in Methods and Fig. 1a.
(b) Photograph of the assembled microscope.
(c) Zoomed-in photograph of **(a)** showing the three Pockels cells used for intensity modulation of each corresponding ultrafast laser, and the broadband and dichroic mirrors used for NIR 2P beam combining.
(d) Zoomed-in photograph of the 1P and 2P light-sheet excitation path, from **(a)**, showing the visible CW lasers, the broadband and dichoric mirrors used for beam combining, the AOTF used to select the 1P wavelengths and to control their amplitudes, as well as the beam conditioning optics used for generating 1P and 2P light sheets.
(e) Zoomed-in photograph of the remote-focus subsystem, from **(a)**, with O2 and O3 oriented at a 45° angle between their optical axes.

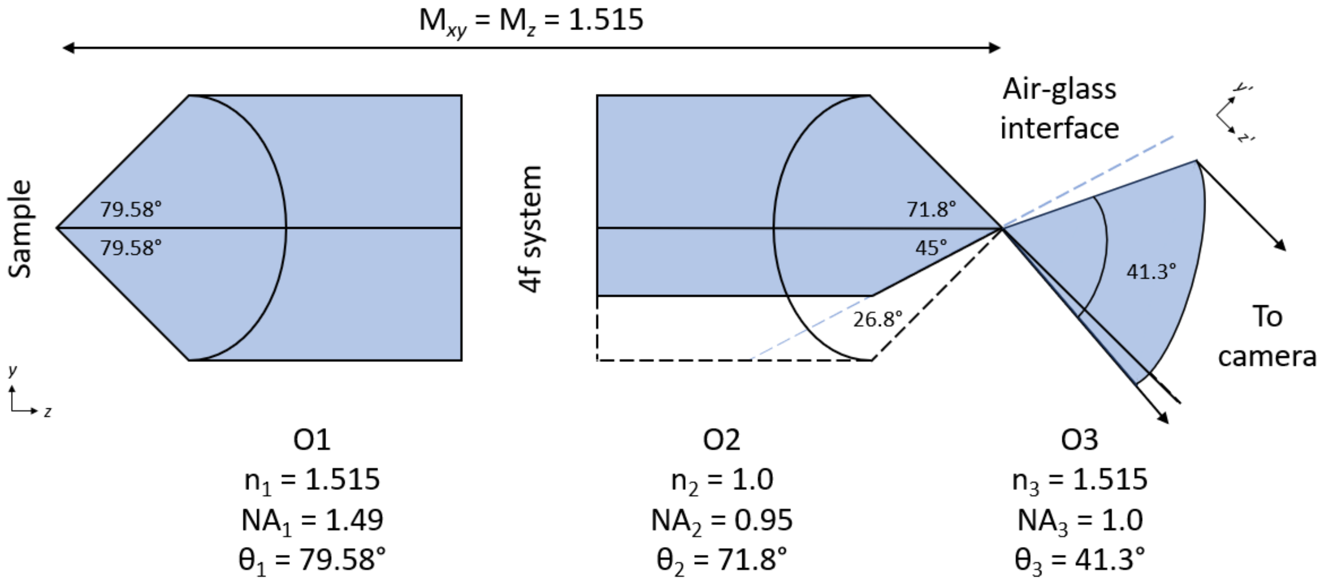

FIG. S2. **Geometric illustration of the theoretical effective detection NA along the y' axis.** Light cones collected by each objective are indicated in blue; black lines represent the paraxial and marginal light rays. The intermediate image at the focal space of O2 is magnified by $NA_1/NA_2 = 1.515\times$ both laterally and axially to minimize aberrations. Nearly all of the light transmitted though O2 is refracted at the air-glass interface (blue dashed line) and enters O3, except for a small portion of light that is clipped (zone between the blue and black dashed lines). Along the x-axis, i.e., normal to the tilt direction, the theoretical effective detection NA is estimated as $1.515 \cdot \sin(71.8^\circ)$, so that 1.44 of O1's nominal NA of 1.49 can be used. Along the tilted y-axis, light is clipped at the air-glass interface; the upper bound for the theoretical effective NA in y is estimated as $1.515 \cdot \sin((71.8^\circ + 45^\circ)/2) = 1.29$.

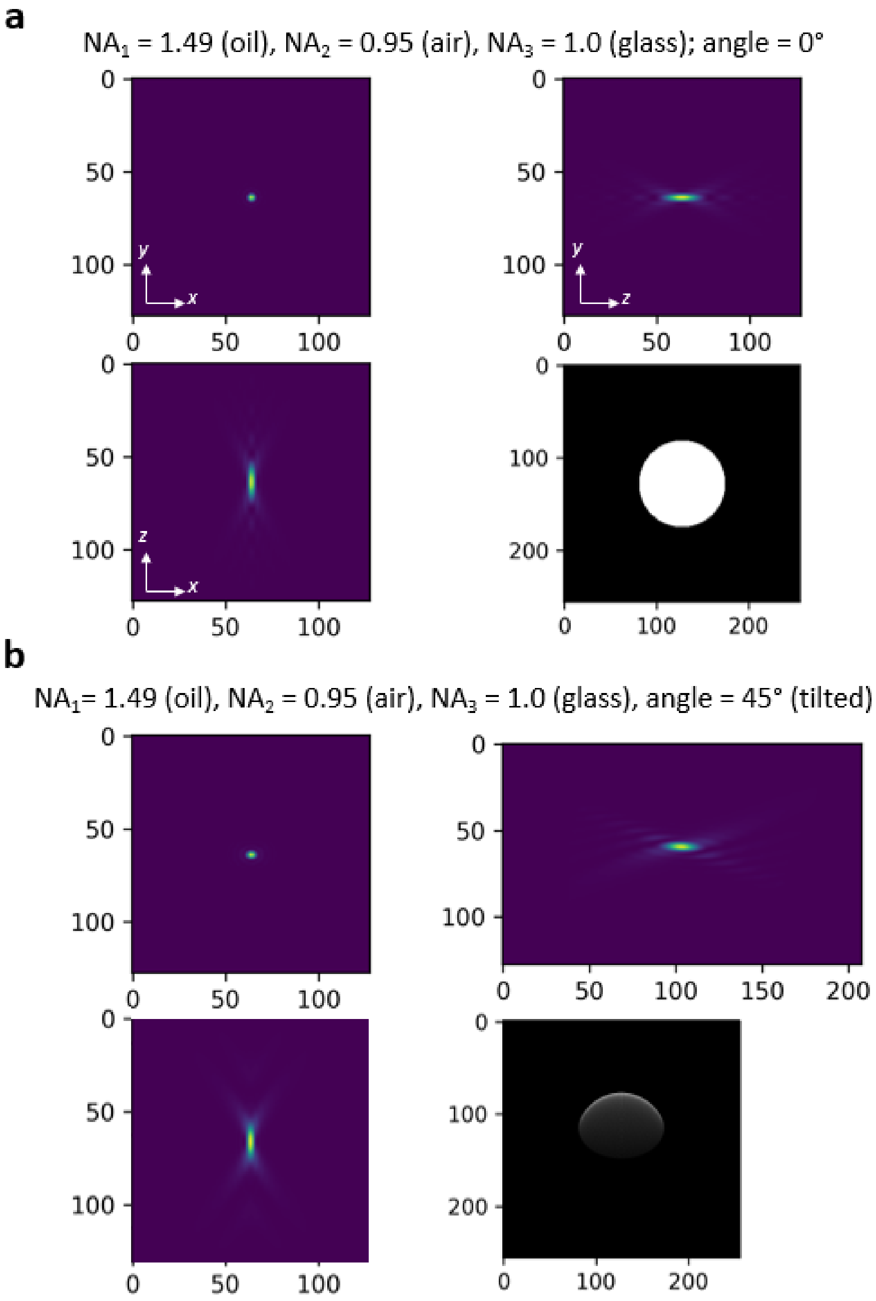

FIG. S3. **Simulations of 2P-OPM detection point spread and pupil functions.**
(a) Simulated xy (top left), xz (bottom left), and yz (top right) cross sections of the PSF and pupil function (botom right) for the high-NA 2P-OPM in straight transmission. The xyz FWHM values of the PSF: 181.8 × 181.8 × 872.3 nm^3
(b) Simulated xy (top left), xz (bottom left), and yz (top right) cross sections of the PSF and pupil function (botom right) for the high-NA 2P-OPM in the tilted configuration. The xyz FWHM values of the PSF with a 45°-tilt: 179.9 × 237.3 × 942.5 nm^3. The FWHM values along x are comparable between the straight and the tilted configurations, suggesting that the full angular aperture of O1 and O2 are utilized. The FWHM values along the other two directions (y and z) are slightly wider in the tilted configuration, due to the asymmetry of the pupil function, and hence the effective resolution is slightly worse.

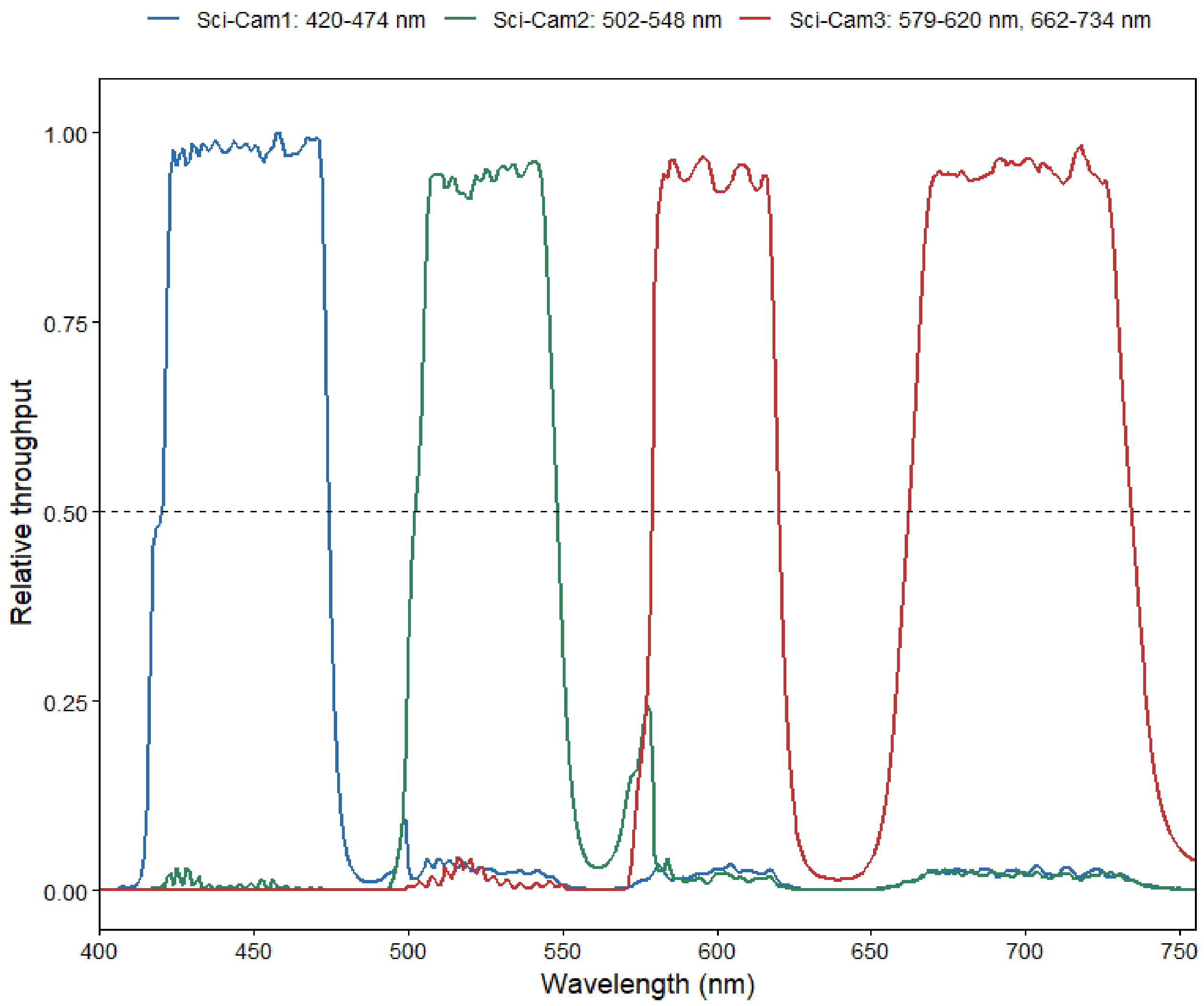

FIG. S4. **Emission channels for 2P-OPM.**
Transmission to each sCMOS camera (Sci-Cam1–3), computed from manufacturer dichroic spectra by multiplying transmissions along the dichroic-mirror tree (reflections taken as R=1−T). Curves share a single global normalization (unitless); the dashed line marks the 50% threshold used to define the passbands shown in the legend. See Table I for parts list and Fig. 1a for optical diagram.

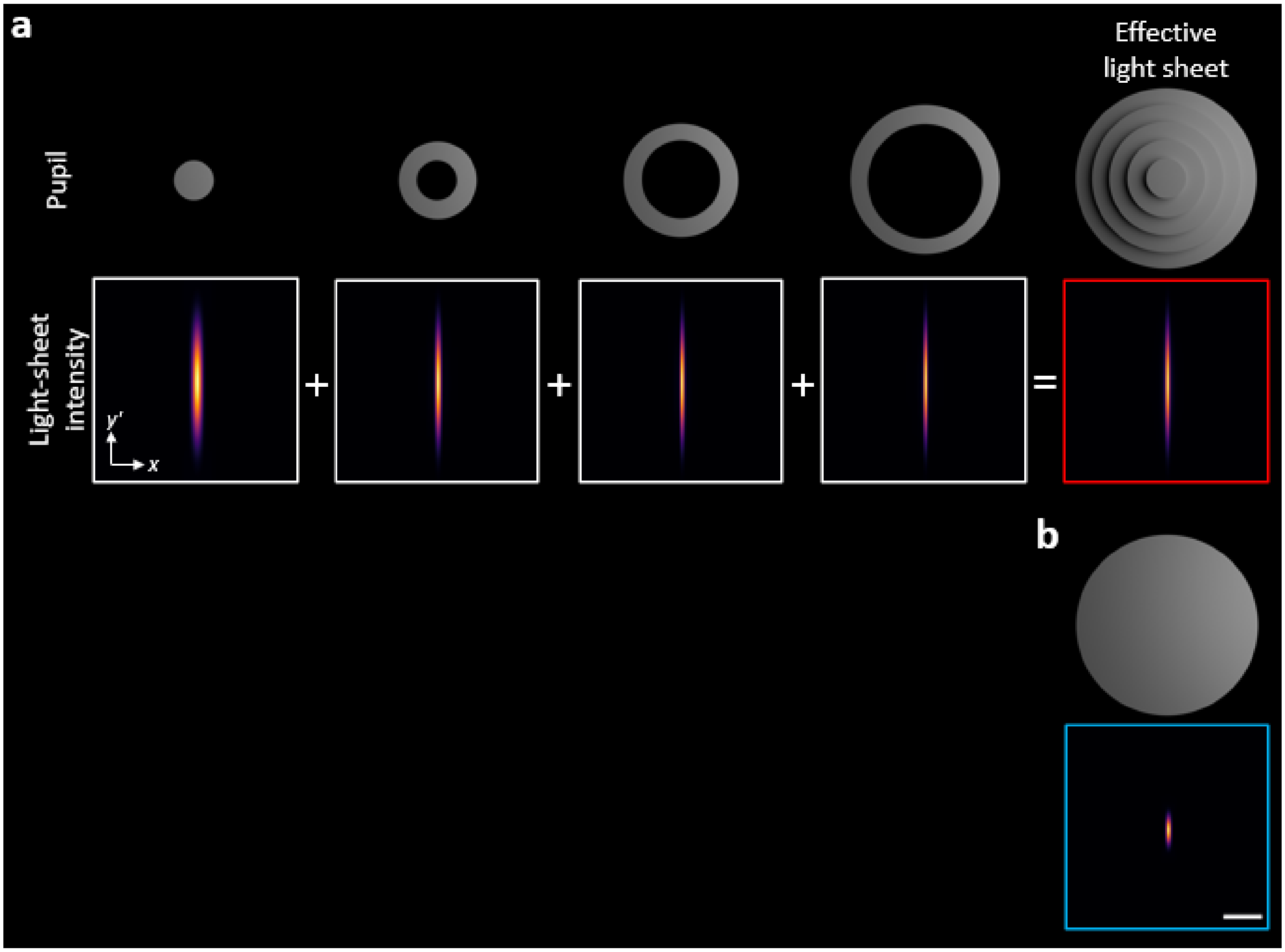

FIG. S5. **Principle and simulations of Bessel-like light sheet.**
(a) A series of apertures at the pupil (top row), each of different diameter, produce the same axial extent (second row). The (left column) aperture produces a Gaussian-like focus, whereas the annular apertures (middle columns) produce a Bessel-like focus. Inspired by the pupil plane approach to extended focus from Gustafsson et al. [60], the layer-cake phase mask (right column) segments the pupil into multiple sub-apertures. The phase mask consists of multiple concentric glass disks, each ∼300 µm thick, creating a time delay between zones longer than the pulse duration of the ultrafast (femtosecond pulse) laser, ensuring negligible temporal overlap and preventing interference between pulses [25, 26]. This results in independent beamlets that add together to produce an axially elongated Bessel-like beam, with minimal broadening to the thickness of the beam focus (right column). The amount of DOF scales linearly to the number of layers in the mask (in this case ∼4×), and can thus be tuned to the desired light-sheet thickness and length.
(b) A conventional high-NA Gaussian beam light sheet, where different wavevectors interfere and add coherently to produce a tight focus, for comparison. Scale bar, 2 µm.

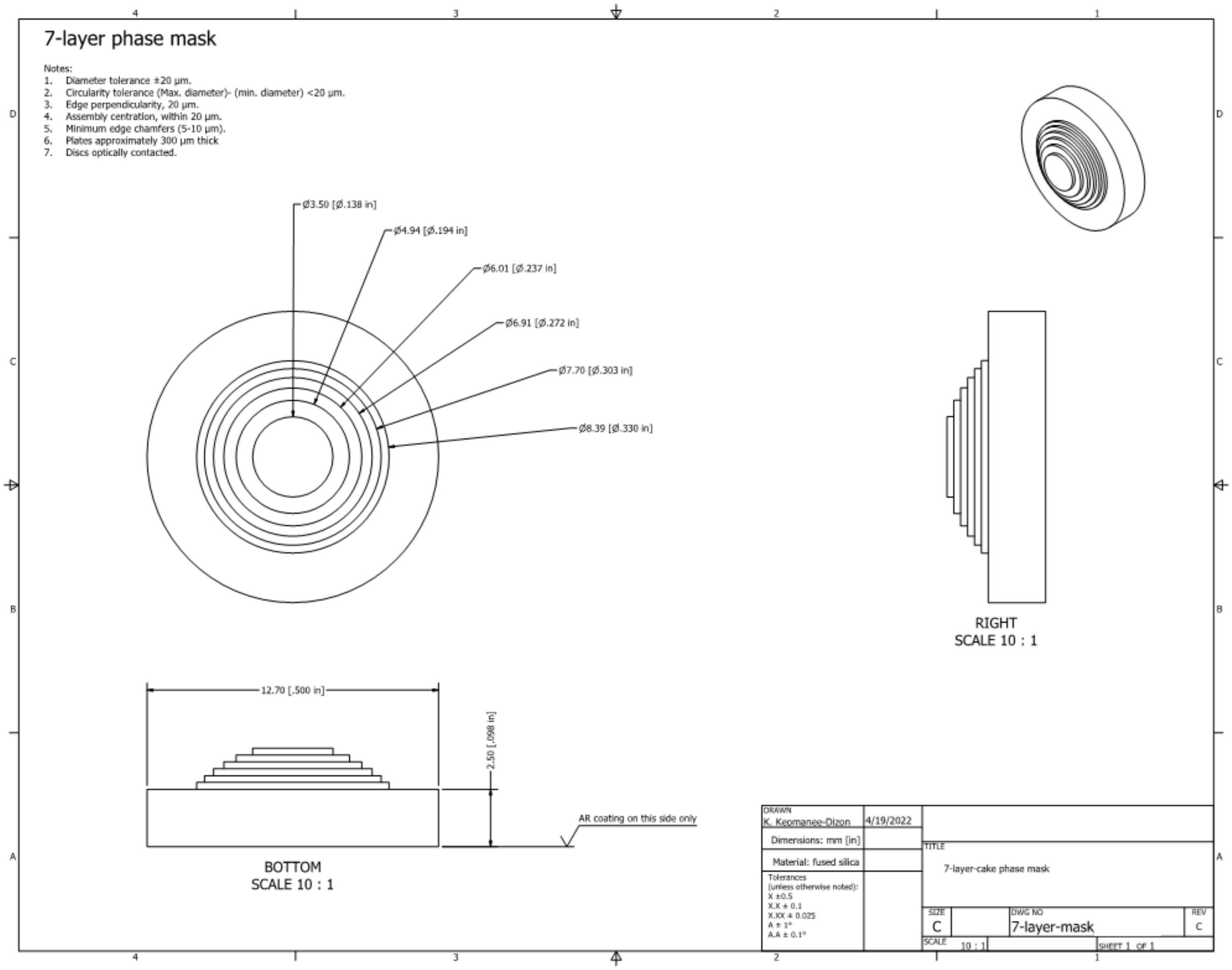

FIG. S6. **Layer-cake phase mask design and drawing.**
In the experiments presented, we under-filled the phase mask and used only 4 of the innermost layers, extending the depth of focus accordingly.

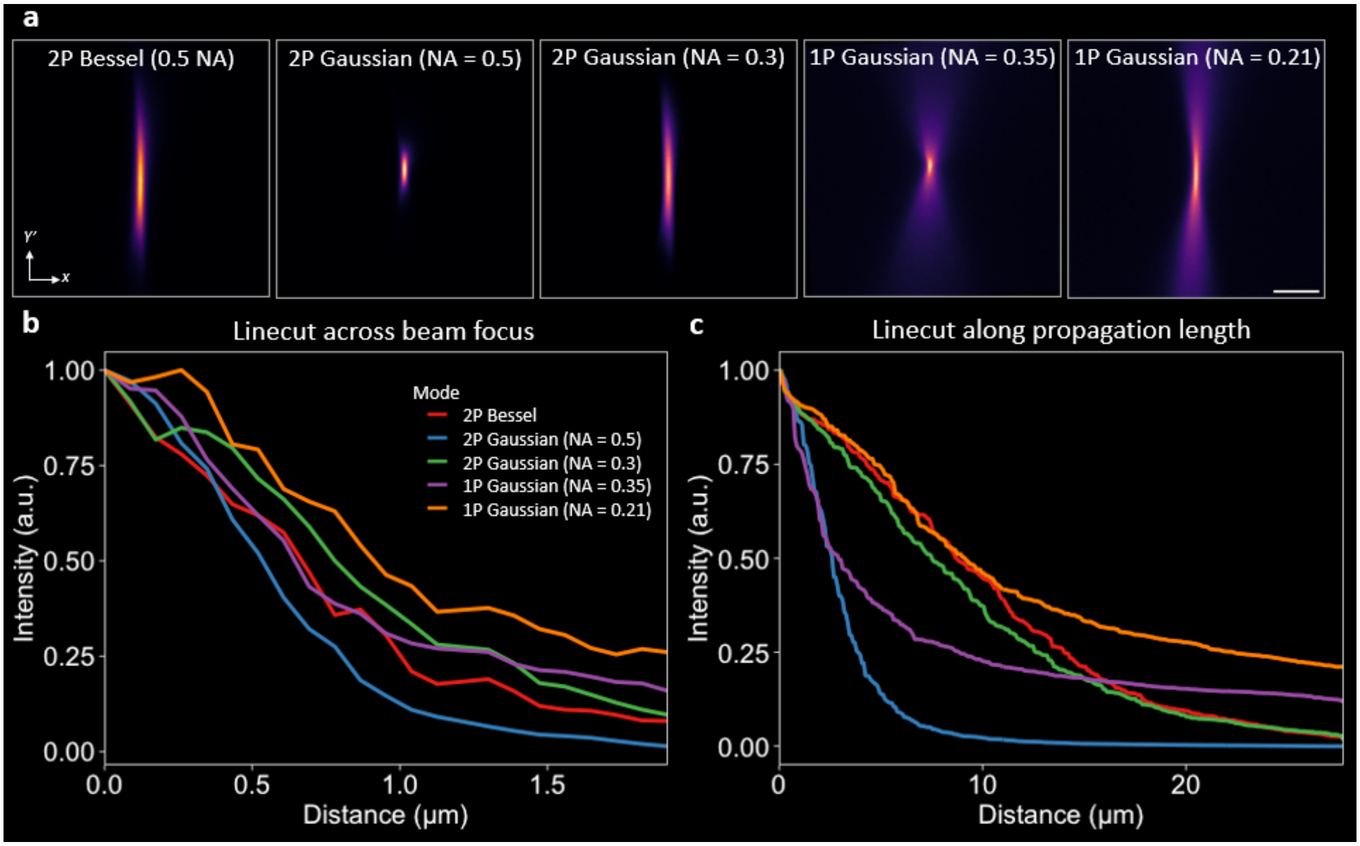

FIG. S7. **Comparison and characterization of light-sheet profiles.**
(a) Experimental images of fluorescence excited by 2P Bessel (column 1), 2P high-NA Gaussian (column 2), 2P low-NA Gaussian (column 3), 1P high-NA Gaussian (column 4), and 1P low-NA Gaussian (column 5) focused beams, which are rapidly scanned in the x direction to create light sheets. Scale bar, 2 µm.
(b) Intensity profiles of **(a)** at the beam focus. The FWHM values are 2P Bessel: ∼0.6 µm; 2P Gaussian high NA: ∼0.58 µm; 2P Gaussian low NA: ∼0.85 µm; 1P Gaussian high NA: ∼0.62 µm; 1P Gaussian low NA: ∼0.95 µm.
(c) Intensity profiles of **(a)** along the propagation length. The FWHM values are 2P Bessel: ∼10 µm; 2P Gaussian high NA: ∼3.2 µm; 2P Gaussian low NA: ∼9.8 µm; 1P Gaussian high NA: ∼3.4 µm; 1P Gaussian low NA: ∼10.2 µm.

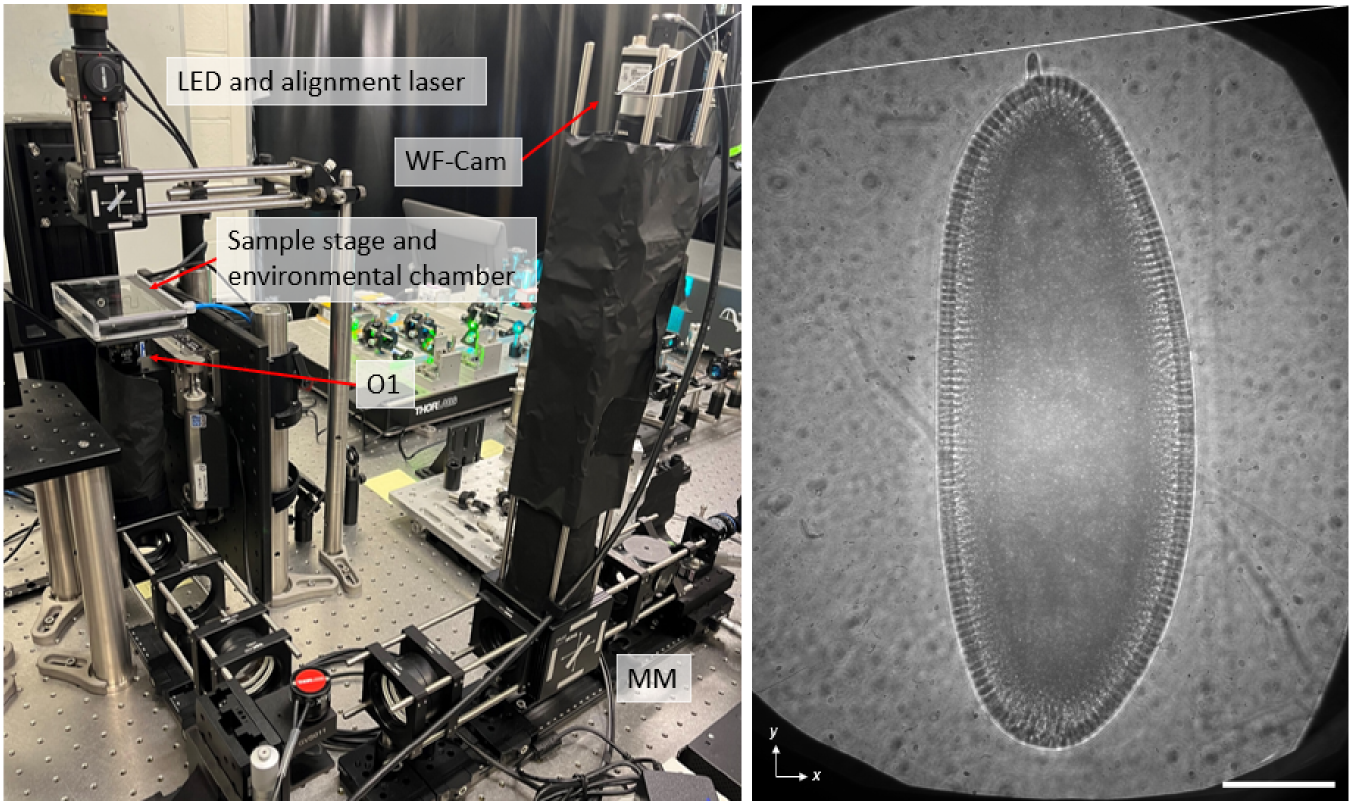

FIG. S8. **Wide-field illumination and detection.**
Photograph of the wide-field illumination module and the wide-field detection camera (WF-Cam) of the 2P-OPM. 455 nm and 625 nm LED light, and/or 405 nm, 488 nm, 561 nm laser light, are used for trans-illumination of the sample (e.g., for photoactivation) and/or alignment of the optical system. A movable mirror (MM) is used to direct light to WF-Cam for specimen view-finding and inspection, recording macroscopic behavior, as well as a photoperturbation indicator. With an effective magnification of 10×, the WF-Cam yields a $> 530 \times 710\ \mu m^2$ field of view. The inset shows an image of a fruit fly embryo, from Video 1. Scale bar, 100 µm.

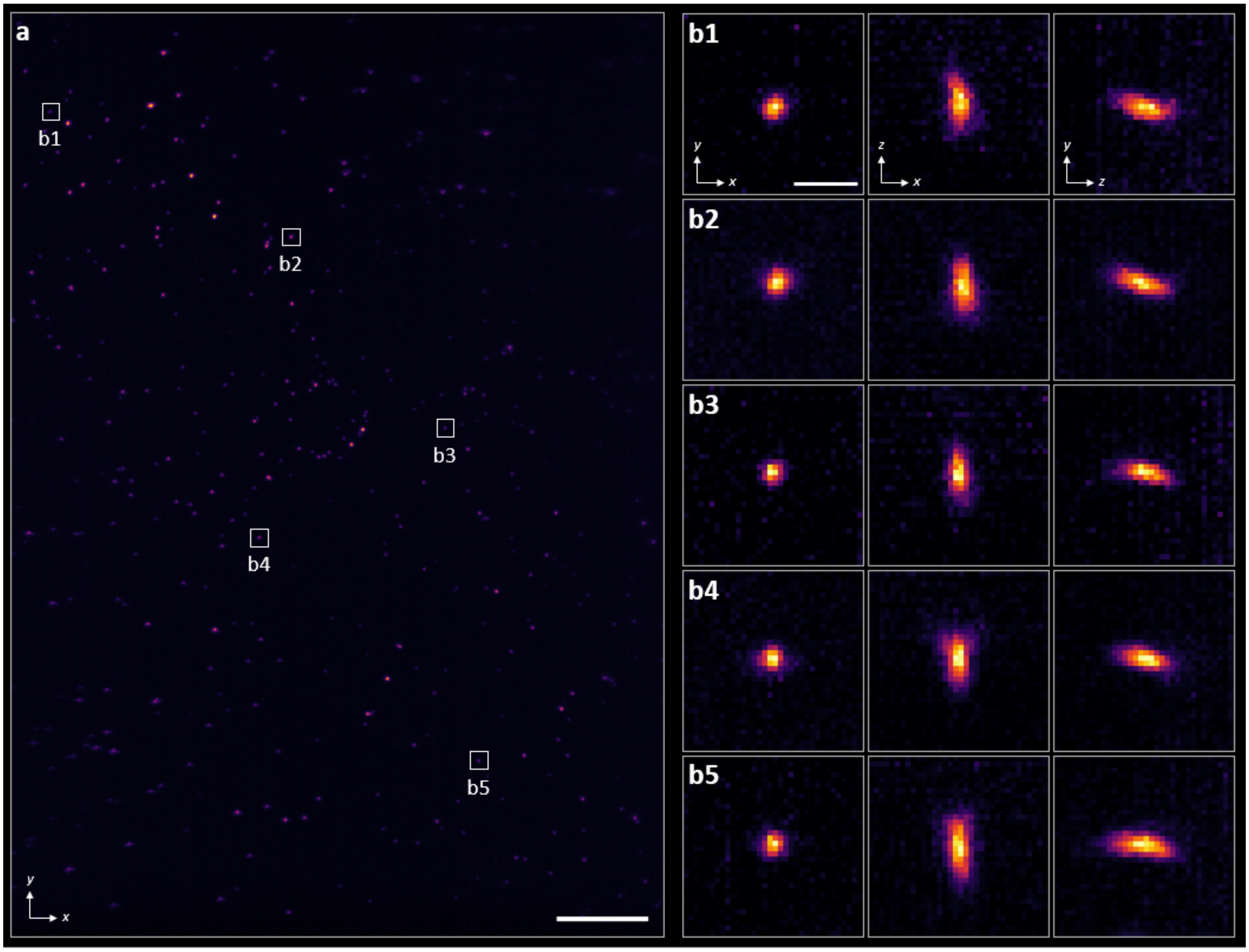

FIG. S9. **2P-OPM resolution.**
(a) xy MIP of a 70 × 100 × 10 (xyz) µm^3 beads field captured with 2P-OPM. Scale bar, 10 µm.
(b1-b5) Zoomed-in regions from **(a)** (left) and corresponding xz (middle) and yz (right) MIPs, showing tight and uniform resolution throughout the volume. Scale bar, 1 µm.

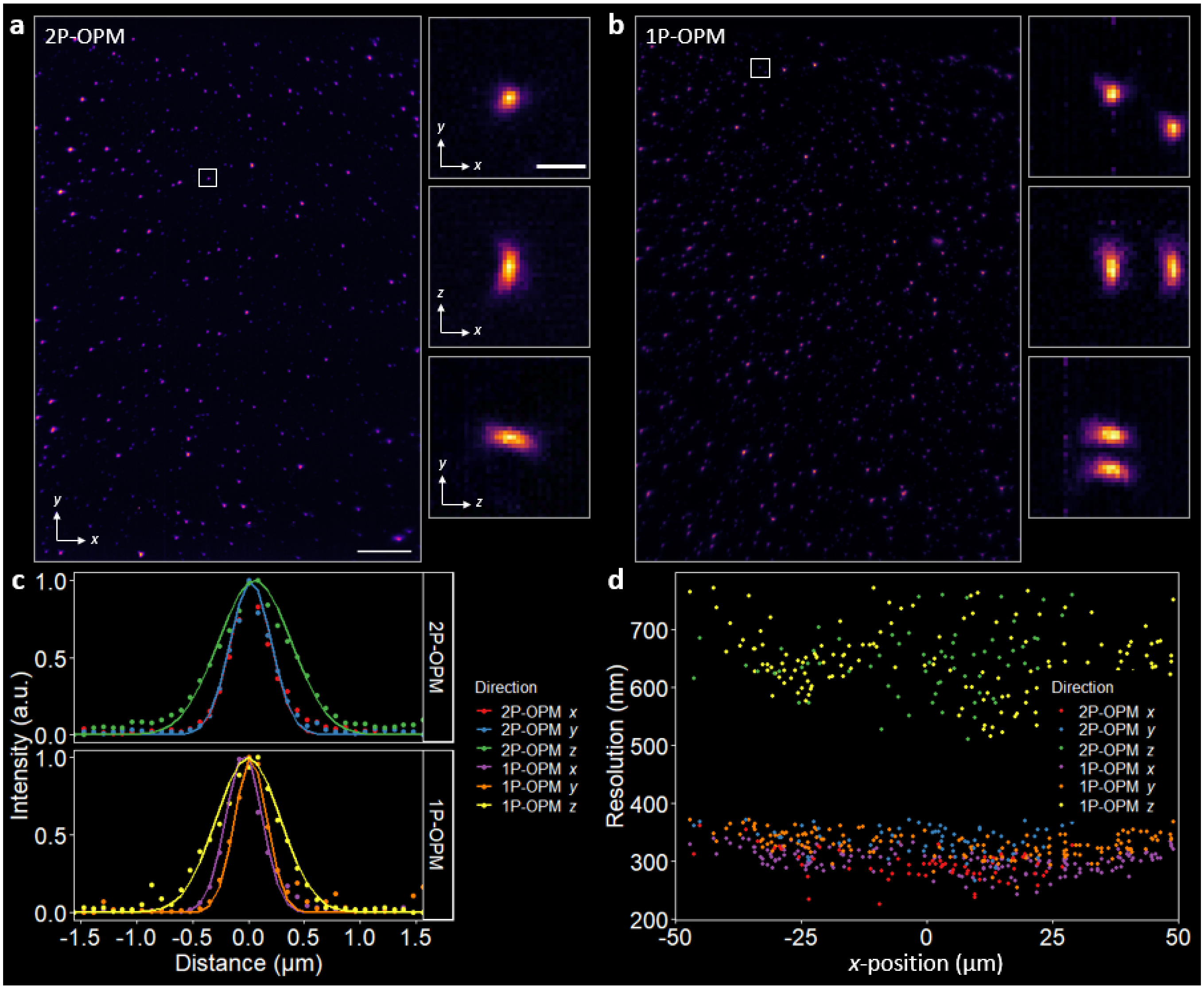

FIG. S10. **2P-OPM PSF benchmarking.**
(a,b) xy MIPs of a 70 × 100 × 15 (xyz) µm^3 volume cut from a beads field captured with 2P-OPM **(a)** and 1P-OPM **(b)**. Scale bar, 10 µm.
Insets show xy (top), xz (middle), and yz (bottom) MIPs of a representative bead from the region indicated by the white box for each respective mode. MIPs use linear contrast, adjusted separately for the fields and insets, so the zoomed views may appear different. Scale bar, 1 µm.
(c) x, y, and z line intensity profiles through the PSFs in the insets of **(a)** and **(b)**, showing comparable resolution in all three dimensions between 2P-OPM and 1P-OPM. Color points: raw data; solid lines: Gaussian fit.
(d) x, y, and z resolution, as measured by the FWHM, across the x-field of view for 2P-OPM and 1P-OPM ($N > 90$ beads for each mode). The mean x, y, and z FWHM ± SD values are 2P-OPM, 292 ± 40 nm, 331 ± 40 nm, 653 ± 84 nm, respectively; and 1P-OPM, 283 ± 27 nm, 324 ± 28 nm, 613 ± 60 nm, respectively.

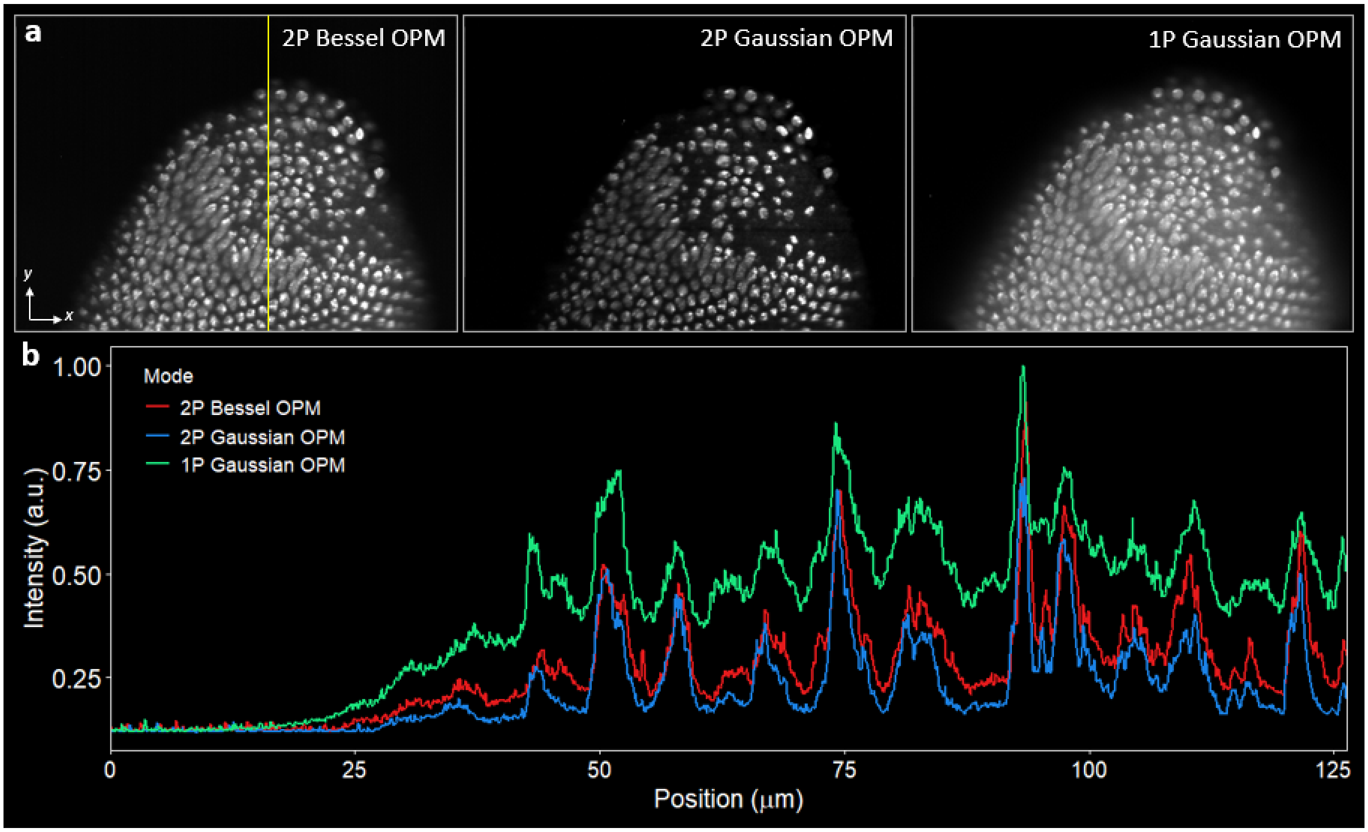

FIG. S11. **Imaging multicellular systems with 2P Bessel, 2P Gaussian, and 1P Gaussian OPM.** **(a)** xy MIP of a DAPI-stained fruit fly embryo captured with 2P Bessel (left), 2P Gaussian (middle), and 1P Gaussian OPM. **(b)** Fluorescence intensity profiles along the yellow line shown in **(a)**. 2P Bessel and 2P Gaussian OPM show progressively improved SNR compared to 1P Gaussian OPM.

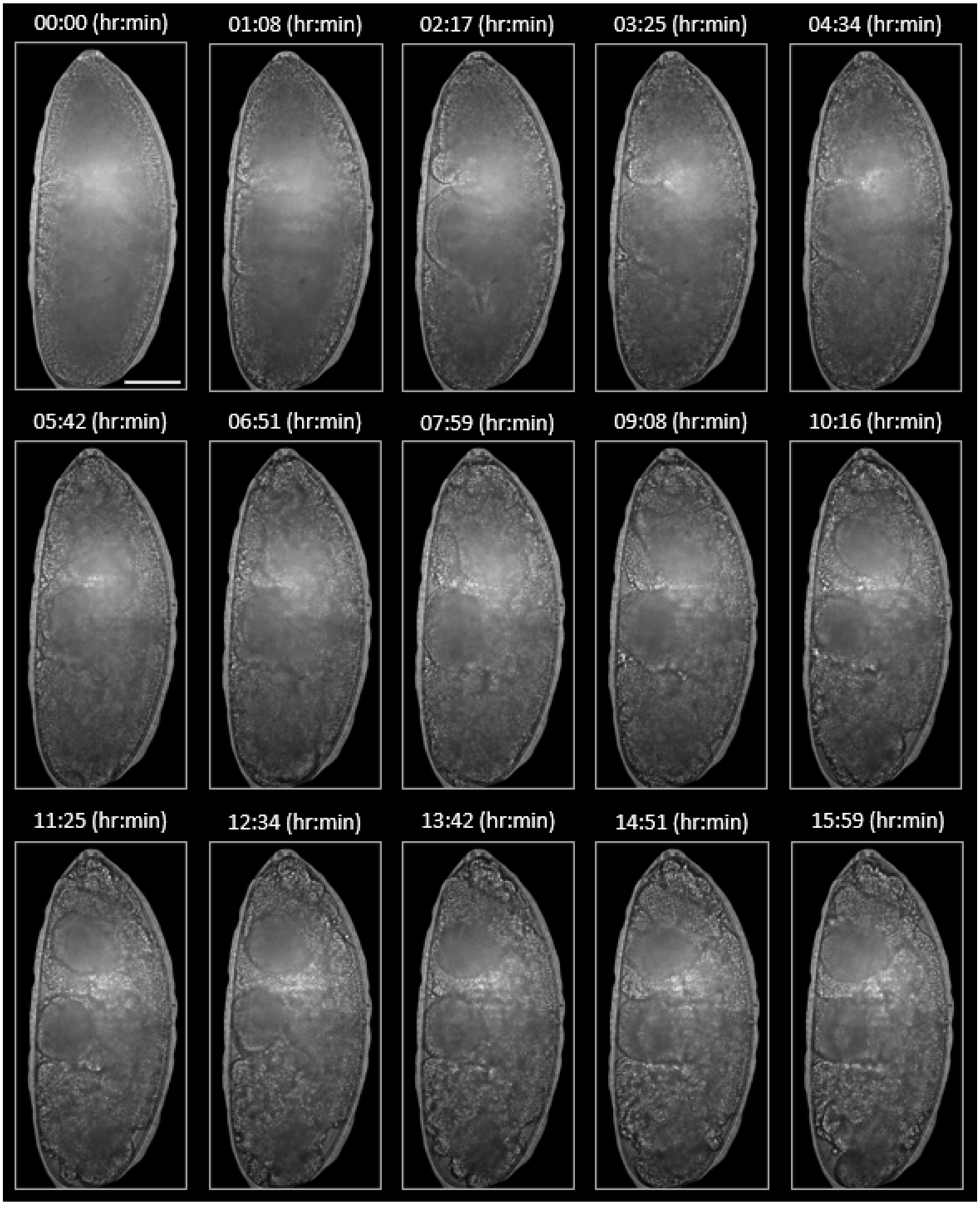

FIG. S12. **16-hr time-lapse of a live *Drosophila* embryo following 2P-OPM imaging.**
Bright-field time-series acquired after 2P-OPM imaging to assess sample viability. 16-hr time-lapse consists of 3000 time points at 20 s intervals. The embryo progressed through normal morphogenesis with no phenotypic signs of phototoxicity throughout the recording. Scale bar, 100 µm.

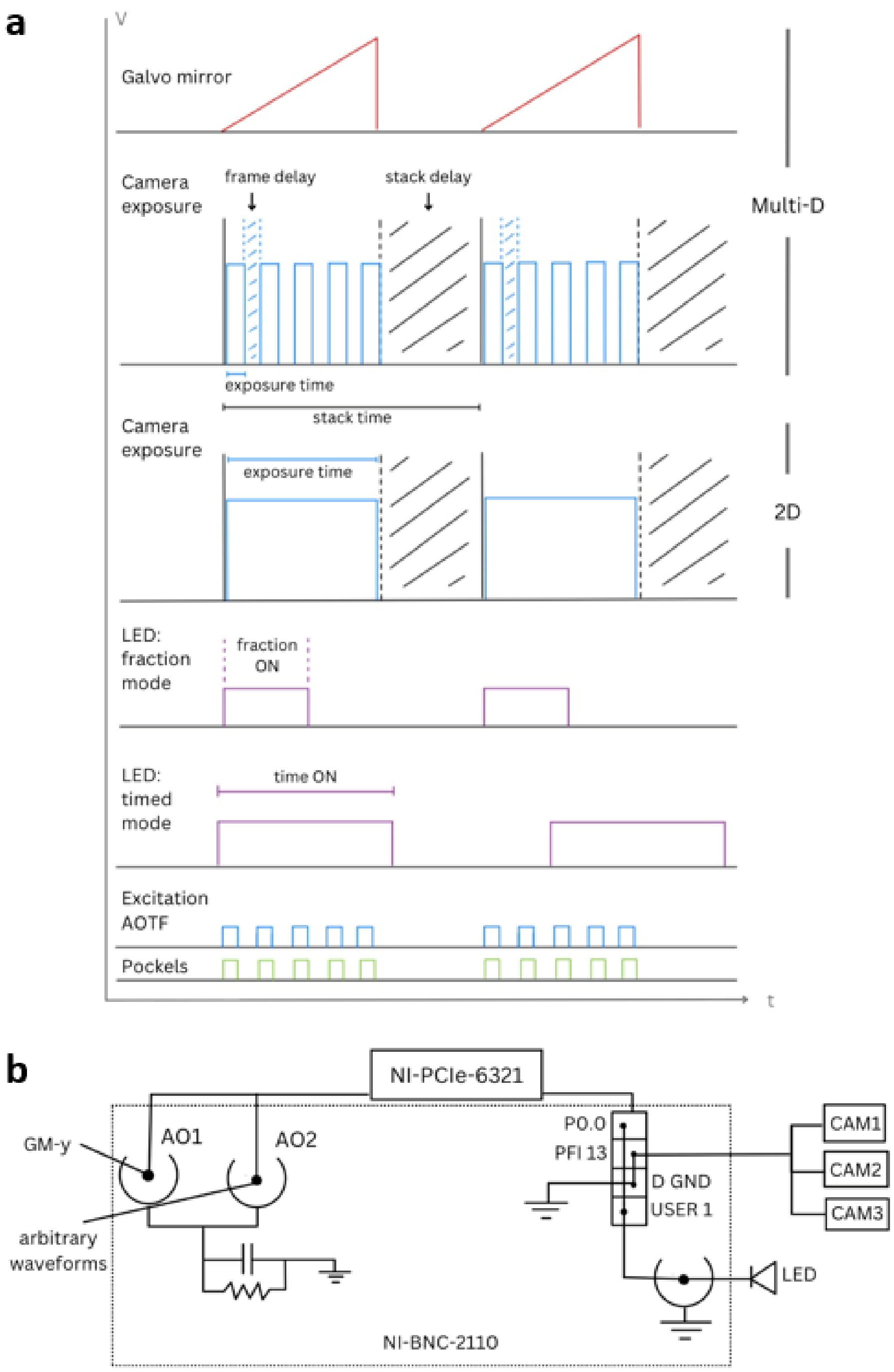

FIG. S13. **Schematic of fastMC control signal sequences and DAQ-device connections.**
(a) Top:GM-y is driven by a sawtooth; the ramp advances planes within a stack and the reset occurs during the stack delay (hatched).
Middle: camera exposure waveforms (blue). In 3D (Multi-D) mode, a burst of exposure pulses forms a y-stack, separated by a stack delay for galvo retrace; in 2D mode, single exposures repeat with a per-frame delay. Cameras run in Exposure Control mode, so each TTL pulse defines both the start and the duration of the exposure; hatched regions indicate readout/idle time.
Lower: Wide-field LED illumination/optogenetic control modes. Fraction mode turns the LED on for a user-defined fraction of each stack; timed mode applies one contiguous on window starting at acquisition.
Bottom traces: The AOTF/Pockels are phase-locked to the exposure pulses; excitation is enabled only during camera exposure and blanked during readout and galvo retrace.
(b) All timing signals in **(a)** are generated by the NI X-Series DAQ under fastMC (see Methods) and wired via the NI-BNC-2110. Analog output ports (AO1, AO2) trigger GM-y to start scanning as defined by the precomputed waveform driven from AO2 (e.g., a signal generator). Digital output ports (PO.0, PFI 13) send concurrent TTL pulses to Sci-Cam–1-3 and the LED. Additional cameras can be added by fanning out the same TTL (provided the acquisition PC can sustain the aggregate throughput).

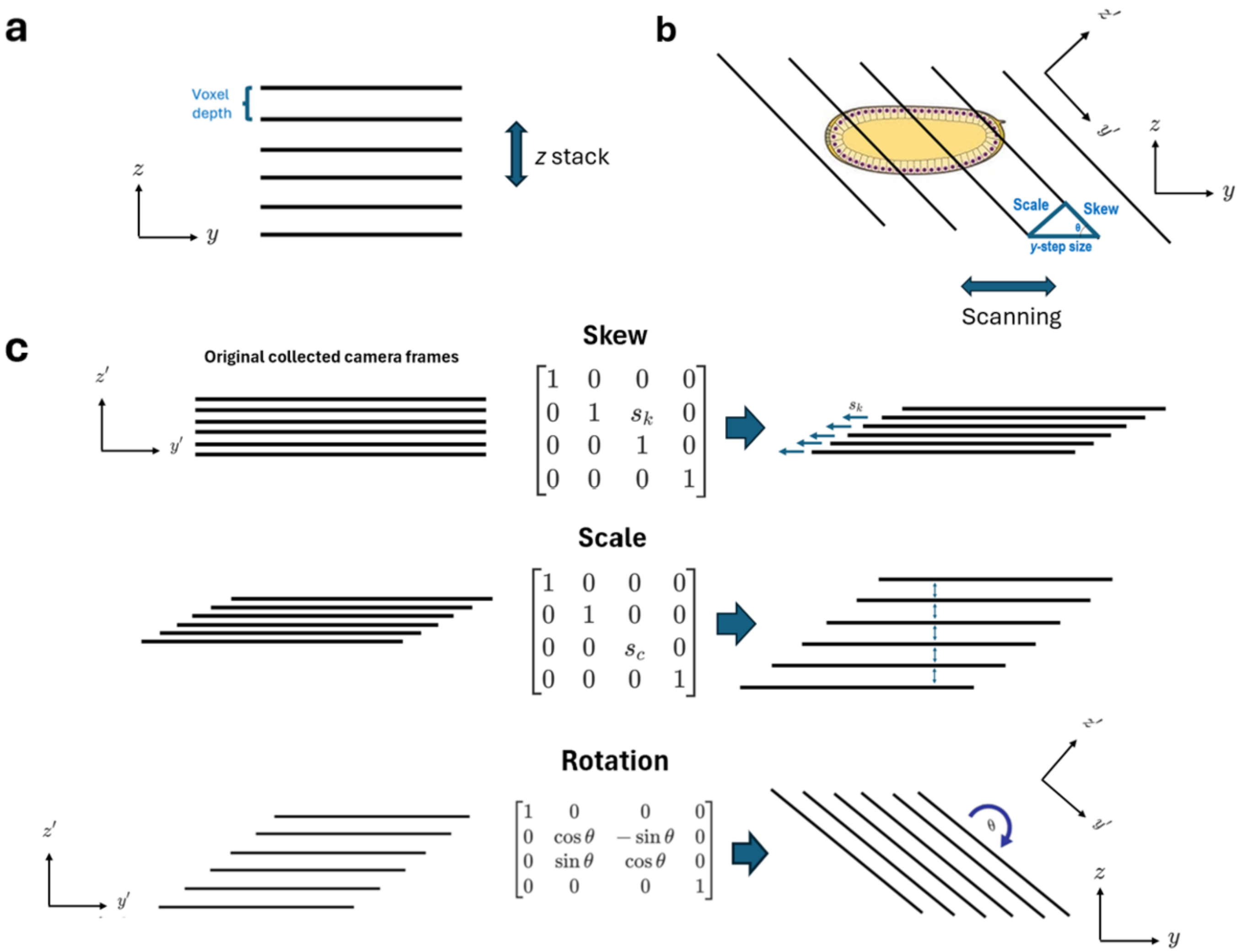

FIG. S14. **OPM geometry and reconstruction.**
(a) Conventional z-stacks.
(b) OPM "y-stacks": tilt by θ produces per-frame offsets $d_{\text{skew}} = y_{\text{step}} \cos\theta$ (along y') and $d_{\text{scale}} = y_{\text{step}} \sin\theta$ (along z'); x and y' are the in-plane camera axes. See also Fig. 1b-d.
(c) Affine sequence *Skew* (K) $\rightarrow$ *Scale* (S) $\rightarrow$ *Rotation* (R) as in Eqs. 4–9, followed by interpolation onto a Cartesian grid (Methods).

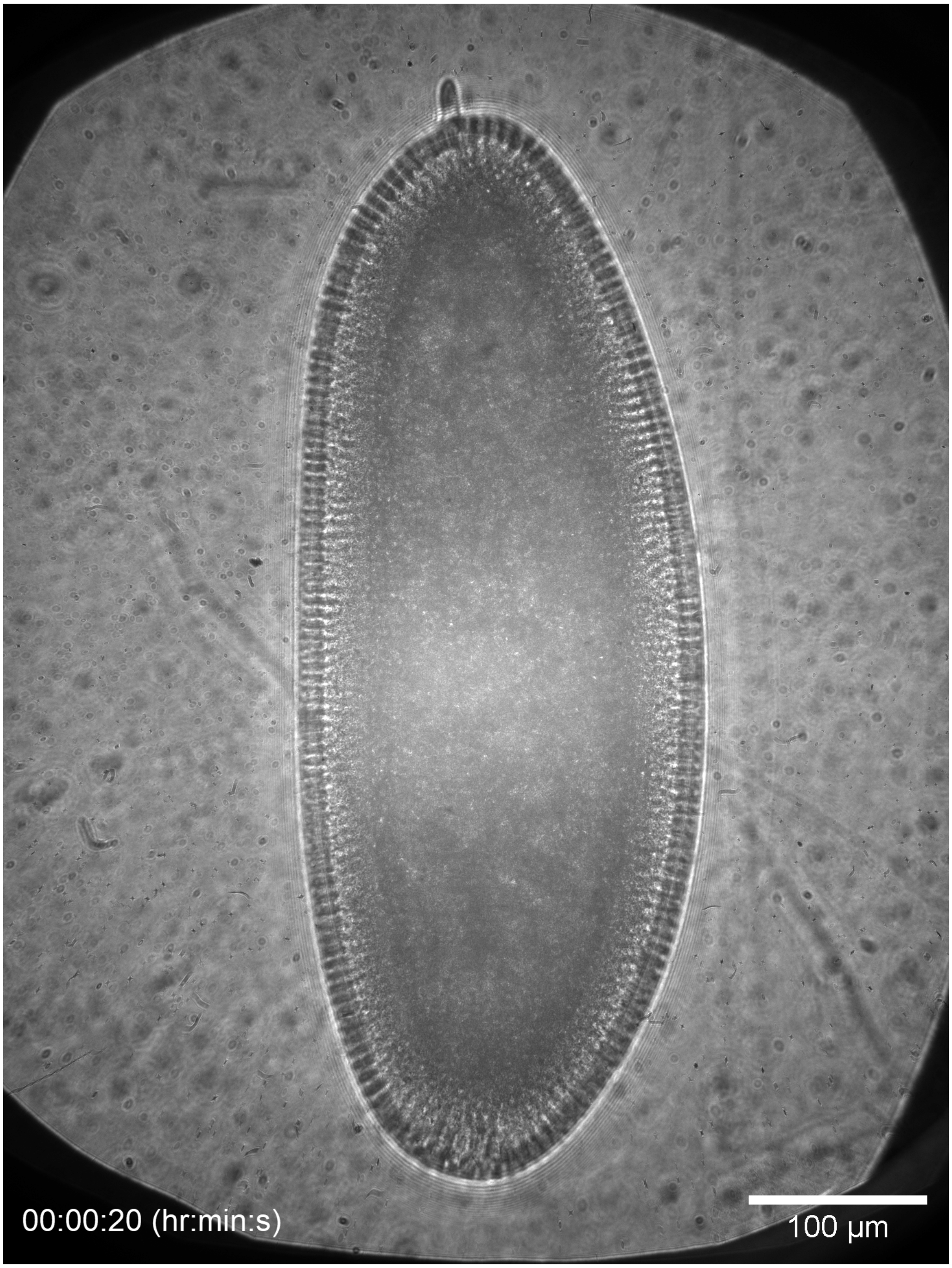

Video 1. **Bright-field movie of *Drosophila* embryogenesis.**
Movie shows normal morphological progression through late embryogenesis. 530 × 710 μm^2 field of view captured at 20 s intervals for ∼21 hrs (3850 time points). The WF-Cam provides a label-free readout that links molecular-scale measurements from the fluorescence cameras (Sci-Cam1–3) to macroscopic behavior, and also serves as a phenotypic indicator for photoperturbation during high-resolution acquisitions.

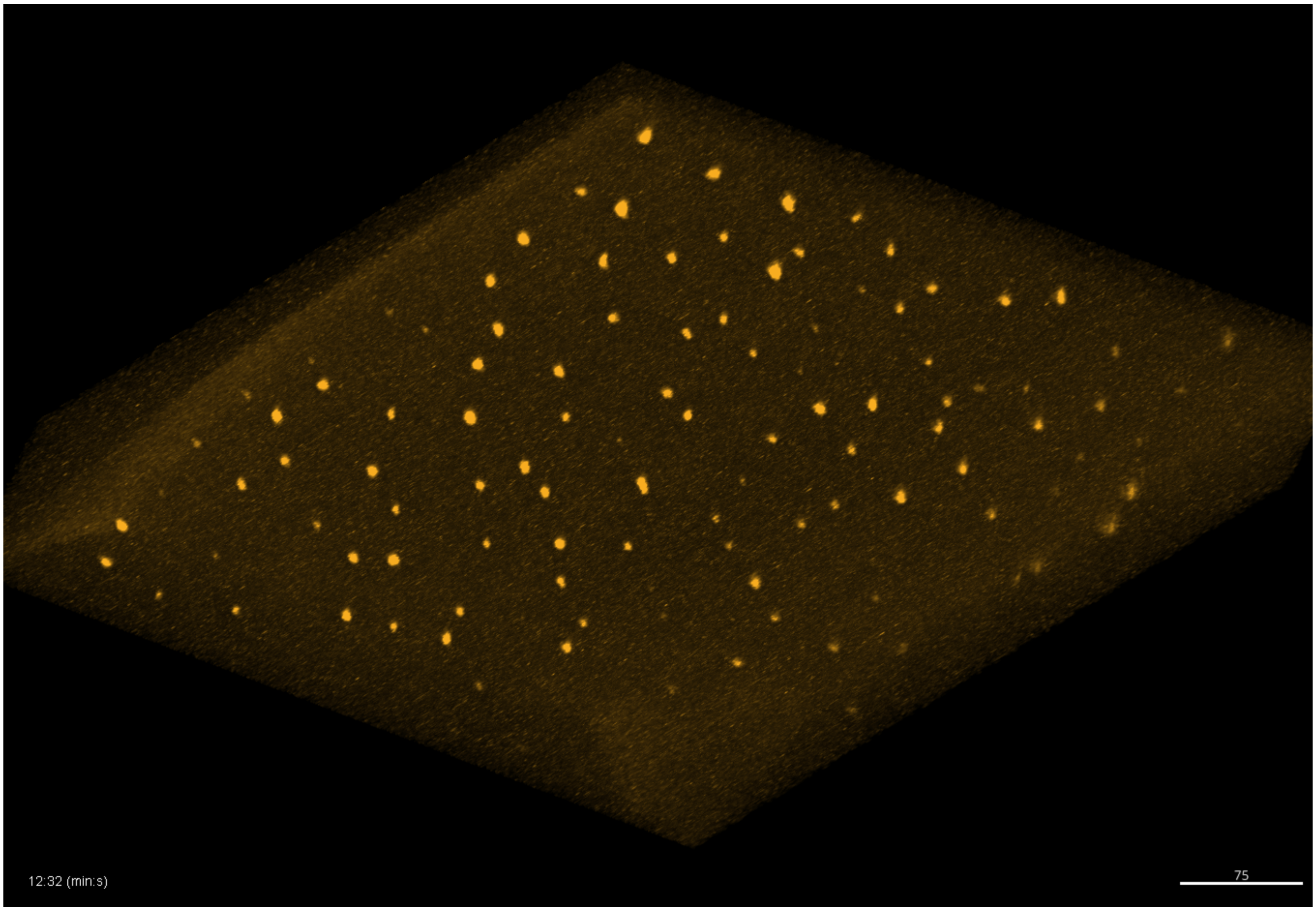

Video 2. **Volumetric time-lapse of transcriptional dynamics during early fly development.**
4D 2P-OPM imaging of active *hb* transcription sites labeled with MS2-MCP-mNeonGreen. 100 × 115 × 15 µm^3 volume recorded at ∼0.05 Hz for ∼46 mins (145 time points). At the first frame (t = 00:00, min:s) the orientation is anterior left, dorsal up; the embryo rotates over time. Scale bar, µm.

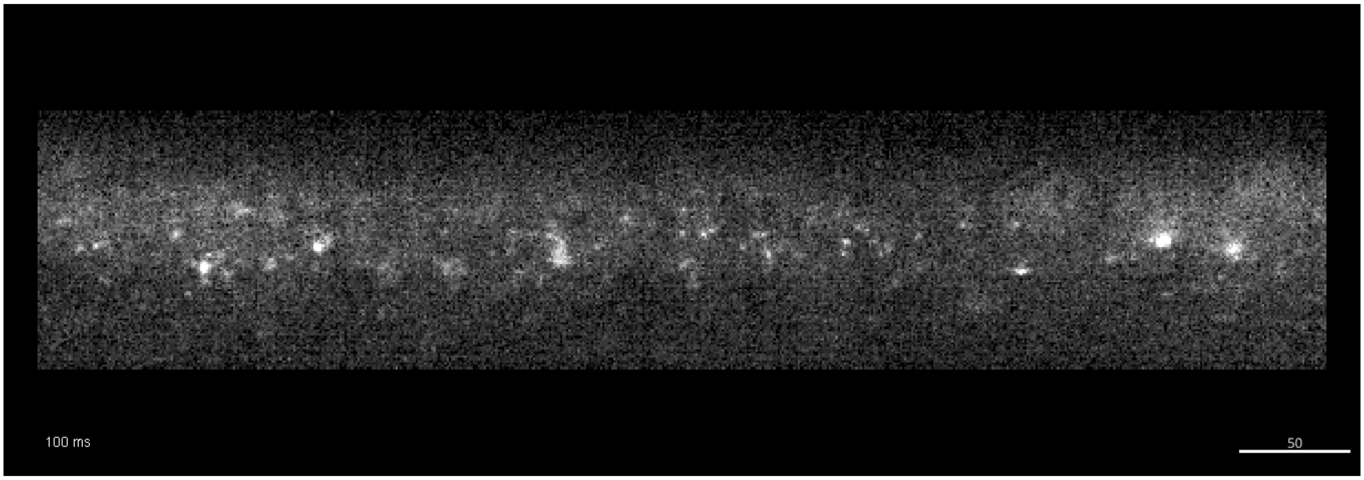

Video 3. **Single-molecule movie of transcripts diffusing in a live fly embryo.**
2P-OPM time-lapse of MS2-MCP-mNeon-labeled *hb* mRNAs showing diffraction-limited spots and brighter transcription sites. 100 × 20 µm^2 field of view acquired at 20 Hz over 50 time points (∼ 2500 ms). The same sequence is replayed ×4 with fly throughs to highlight zoomed subregions. Scale bar, µm.

TABLE SI. **Imaging conditions.**

**Figure and Video**	**Mode**	**Sample**	**Fluorescent label**	**Temperature**	**Excitation** λ (nm), NA, Power† (mW)	**Voxel** a_x, a_y, a_z (nm)	***y* step (nm)**	**Volume** x, y, z (µm)	**Exposure** (ms)	**Emission channel**	$\Delta t \times N$¶ (Time interval (s) × Time points)
Fig. 2a,b and S10a,c,d	2P-OPM	50 nm beads	Dragon Green	Room	925, 0.3, 464	87, 87, 87	87	100, 100, 15	500	Sci-Cam2	577 × 1
Fig. 2b and S10b–d	1P-OPM	50 nm beads	Dragon Green	Room	488, 0.21, 0.2	87, 87, 87	87	100, 100, 15	75	Sci-Cam2	86.55 × 1
Fig. 3a	Wide-field	mESC gastruloid	N/A	Room	625, WF§, 45‡	345, 345	N/A	530, 710	1500	WF-Cam	1.5 × 1
Fig. 3a–d	2P-OPM	mESC gastruloid	DAPI	Room	750, 0.3, 128	87, 87, 87	347	100, 115, 15	300	Sci-Cam1	86.7 × 1
Fig. 3a–d	1P-OPM	mESC gastruloid	DAPI	Room	405, 0.21, 0.14	87, 87, 87	347	100, 115, 15	300	Sci-Cam1	86.7 × 1
Fig. 3e	Wide-field	mESC gastruloid	N/A	Room	625, WF§, 45‡	345, 345	N/A	530, 710	1500	WF-Cam	1.5 × 1
Fig. 3e–h	2P-OPM	mESC gastruloid	FOXC1-Alexa Fluor 546	Room	1030, 0.18, 33	87, 87, 87	347	71, 130, 30	500	Sci-Cam3	144.5 × 1
Fig. 3e–h	1P-OPM	mESC gastruloid	FOXC1-Alexa Fluor 546	Room	561, 0.12, 0.08	87, 87, 87	347	71, 130, 30	500	Sci-Cam3	144.5 × 1
Fig. 4a–c	2P-OPM	Fly, NC 14	*eve*-ATTO 565	Room	1030, 0.3, 38	87, 87, 87	173	100, 115, 15	500	Sci-Cam3	289.5 × 1
Fig. 4a–c	1P-OPM	Fly, NC 14	*eve*-ATTO 565	Room	561, 0.21, 0.08	87, 87, 87	173	100, 115, 15	500	Sci-Cam3	289.5 × 1
Fig. 5a	Wide-field	Fly, NC 14	N/A	Room	625, WF§, 45‡	345, 345	N/A	530, 710	1500	WF-Cam	1.5 × 1
Fig. 5a	2P-OPM	Fly, NC 14	Bcd-eGFP<br>*hb*-MS2-MCP-mCherry	Room	920, 0.3, 200<br>1030, 0.3, 60	173, 173, 173	347	100, 115, 15	800	Sci-Cam2<br>Sci-Cam3	154.4 × 10
Fig. 5b,c and Video 2	2P-OPM	Fly, NC 12	*hb*-MS2-MCP-mNeonGreen	Room	920, 0.3, 80	173, 173, 173	520	100, 105, 15	100	Sci-Cam2	19.3 × 145
Fig. 5d	2P-OPM	Fly, NC 12	*hb*-MS2-MCP-mNeonGreen	Room	920, 0.3, 80	173, 173, 173	520	100, 115, 15	50	Sci-Cam2	9.65 × 398
Fig. 5e–h and Video 3	2P-OPM	Fly, NC 14	*hb*-MS2-MCP-mNeonGreen	Room	920, 0.3, 122	173, 173	N/A	100, 20	50	Sci-Cam2	0.05 × 50
Fig. 6a–d	2P-OPM<br>Photoactivation	RPE-1 OptoEGFR cells	FusionRed-OptoEGFR	37 °C	1030, 0.3, 10<br>455, WF§, 120‡	87, 87, 87	347	50, 65, 15	1000	Sci-Cam3	1200 × 2<br>90 × 14
Fig. S8 and Video 1	Wide-field	Fly, NC14	N/A	Room	625, WF§, 45‡	345, 345	N/A	530, 710	1500	WF-Cam	20 × 3850
Fig. S9a–b	2P-OPM	50 nm beads	Dragon Green	Room	925, 0.3, 590	87, 87, 87	87	100, 100, 10	300	Sci-Cam2	346.2 × 1
Fig. S11a,b	2P Bessel OPM	Fly, NC 14	DAPI	Room	750, 0.5, 106	87, 87, 87	200	180, 130, 15	400	Sci-Cam1	356.4 × 1
Fig. S11a,b	2P-OPM	Fly, NC 14	DAPI	Room	750, 0.5, 8	87, 87, 87	200	180, 130, 15	400	Sci-Cam1	356.4 × 1
Fig. S11a,b	1P-OPM	Fly, NC 14	DAPI	Room	405, 0.35, 0.28	87, 87, 87	200	180, 130, 15	400	Sci-Cam1	356.4 × 1
Fig. S12	Wide-field	Fly, stage 6	N/A	Room	625, WF§, 45‡	345, 345	N/A	530, 710	1500	WF-Cam	20 × 3000

† Unless noted, powers were measured at the back aperture of O1.

‡ LED/photoactivation powers were measured at the sample.

§ WF: Transmitted wide-field illumination was ~10 mm in diameter at the sample, overfilling O1's field of view.

¶ Δt: time between consecutive frames (2D) or volumes (3D). For continuous acquisition, Δt = acquisition time per frame/volume; if a pause is added, Δt includes that pause. N: frames or volumes.
